# Donor T cell STAT3 deficiency enables tissue PD-L1–dependent prevention of graft-versus-host disease while preserving graft-versus-leukemia activity

**DOI:** 10.1172/JCI165723

**Published:** 2023-08-01

**Authors:** Qinjian Li, Xiaoqi Wang, Qingxiao Song, Shijie Yang, Xiwei Wu, Dongyun Yang, Isabelle J. Marié, Hanjun Qin, Moqian Zheng, Ubaydah Nasri, Xiaohui Kong, Bixin Wang, Elizabeth Lizhar, Kaniel Cassady, Josh Tompkins, David Levy, Paul J. Martin, Xi Zhang, Defu Zeng

**Affiliations:** 1 Medical Center of Hematology, Xinqiao Hospital, State Key Laboratory of Trauma, Burn and Combined Injury, Army Medical University, Chongqing, China.; 2 Arthur D. Riggs Diabetes and Metabolism Research Institute, The Beckman Research Institute of City of Hope, Duarte, California, USA.; 3 Hematologic Malignancies and Stem Cell Transplantation Institute, City of Hope National Medical Center, Duarte, California, USA.; 4 Fujian Medical University Center of Translational Hematology, Fujian Institute of Hematology, and Fujian Medical University Union Hospital, Fuzhou, China.; 5 Department of Computational and Quantitative Medicine, Beckman Research Institute of City of Hope, Duarte, California, USA.; 6 Department of Pathology, NYU Grossman School of Medicine, New York, USA.; 7 Fred Hutchinson Cancer Center, Seattle, Washington, USA.

**Keywords:** Hematology, Transplantation, Adaptive immunity, Bone marrow transplantation, T cells

## Abstract

STAT3 deficiency (STAT3^–/–^) in donor T cells prevents graft-versus-host disease (GVHD), but the impact on graft-versus-leukemia (GVL) activity and mechanisms of GVHD prevention remains unclear. Here, using murine models of GVHD, we show that STAT3^–/–^ donor T cells induced only mild reversible acute GVHD while preserving GVL effects against nonsusceptible acute lymphoblastic leukemia (ALL) cells in a donor T cell dose–dependent manner. GVHD prevention depended on programmed death ligand 1/programmed cell death protein 1 (PD-L1/PD-1) signaling. In GVHD target tissues, STAT3 deficiency amplified PD-L1/PD-1 inhibition of glutathione (GSH)/Myc pathways that regulate metabolic reprogramming in activated T cells, with decreased glycolytic and mitochondrial ATP production and increased mitochondrial ROS production and dysfunction, leading to tissue-specific deletion of host-reactive T cells and prevention of GVHD. Mitochondrial STAT3 deficiency alone did not reduce GSH expression or prevent GVHD. In lymphoid tissues, the lack of host-tissue PD-L1 interaction with PD-1 reduced the inhibition of the GSH/Myc pathway despite reduced GSH production caused by STAT3 deficiency and allowed donor T cell functions that mediate GVL activity. Therefore, STAT3 deficiency in donor T cells augments PD-1 signaling–mediated inhibition of GSH/Myc pathways and augments dysfunction of T cells in GVHD target tissues while sparing T cells in lymphoid tissues, leading to prevention of GVHD while preserving GVL effects.

## Graphical abstract

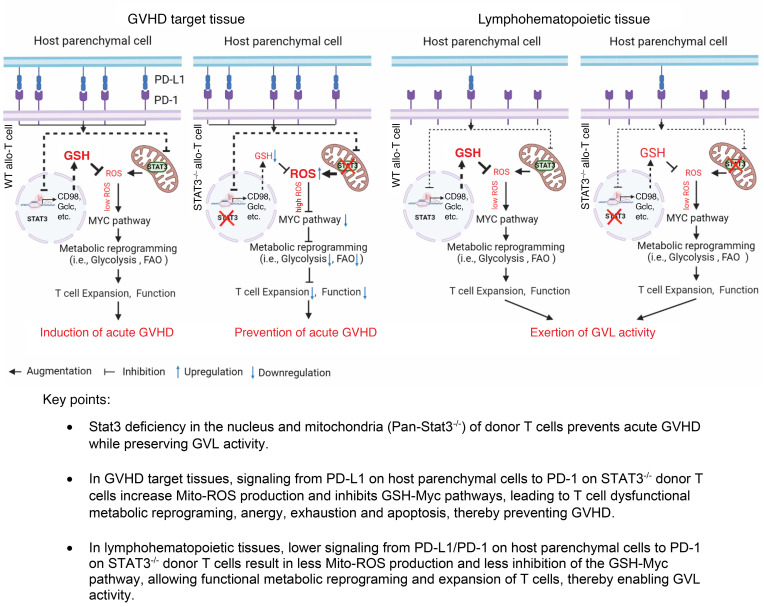

## Introduction

Allogeneic hematopoietic cell transplantation (Allo-HCT) is a curative therapy for relapsed hematological malignances (i.e., leukemia and lymphoma) owing to the graft-versus-leukemia (GVL) activity mediated by alloreactive T cells, but the same alloreactive T cells also mediate graft-versus-host disease (GVHD) (1–5). GVHD is an overexaggerated immune response cascade initiated by activation of alloreactive T cells by host antigen-presenting cells (APCs), such as dendritic cells. The alloreactive donor T cells infiltrating GVHD target tissues (i.e., gut, liver, lung, and skin) cause tissue damage through a variety of mechanisms (6–9). Preventing acute GVHD while preserving GVL activity remains a long-sought goal.

We and others have shown that tissue programmed death ligand 1/programmed cell death protein 1 (PD-L1/PD-1) signaling effectively reduced acute GVHD severity caused by donor CD4^+^ T or CD8^+^ T cells alone, but not by CD4^+^ and CD8^+^ T cells together (10, 11). T cells from STAT3-deficient (STAT3^–/–^) donors, however, did not cause GVHD, even when donor CD4^+^ and CD8^+^ T cells were both present in the graft (12–14). IL-2 from CD4^+^ T cells makes the CD4^+^ and CD8^+^ T cells resistant to induction of anergy, exhaustion, and apoptosis by PD-L1/PD-1 signaling (10, 15, 16), and IL-2 also augmented activation of STAT3 and enhanced glycolysis in T cells (17, 18). It was suggested that PD-1 signaling augments production of mitochondrial reactive oxygen species (Mito-ROS) because pathogenic alloreactive T cells in GVHD target tissues were PD-1^hi^ROS^hi^ (19, 20), although PD-1 signaling also reduces Mito-ROS production by activated T cells in vitro (21). Phosphorylation of STAT3 on Serine 727 (S^727^) enables pSTAT3 entry into mitochondria (Mito-STAT3) (22). While Mito-STAT3 augmented ATP production and reduced ROS production, Mito-STAT3 deficiency reduced mitochondrial ATP (Mito-ATP) production and increased Mito-ROS production, leading to apoptosis in certain cancer cells (23–26). The effects of interactions between STAT3 deficiency and PD-1 signaling on metabolism and ROS production in activated T cells and the subsequent development of anergy, exhaustion, and apoptosis of donor T cells in Allo-HCT recipients have not been elucidated. It also remains unclear whether Mito-STAT3 deficiency alone or increased Mito-ROS alone is sufficient to prevent GVHD.

T cell activation and expansion require metabolic reprogramming of glycolysis and oxidative phosphorylation (OXPHOS) (27–29). Previous reports have suggested conflicting conclusions regarding the respective contributions of glycolysis and OXPHOS in alloactivated T cells that cause GVHD (30–34). A recent study showed that the glutathione (GSH)/Myc pathway has a key role in priming T cell metabolic reprogramming for tissue inflammation (35). Upon activation, T cells produce ROS that triggers an antioxidative GSH response to protect against cellular damage. GSH buffering of ROS also supports activation of the mTOR/NFAT/Myc pathway that enhances glycolysis and glutaminolysis in activated T cells, resulting in T cell proliferation and cytokine production and tissue inflammation. Reduction of GSH production by catalytic subunit of glutamate cysteine ligase (GCLC) deficiency in T cells impairs their function and prevents their ability to cause experimental autoimmune encephalomyelitis (35). These observations raise the question of whether STAT3 deficiency prevents GVHD by inhibiting the GSH/Myc pathway.

In the current studies, we dissected the mechanisms that explain how PD-1 signaling interacts with STAT3 deficiency in donor T cells to prevent GVHD while maintaining GVL activity in a murine model of MHC-mismatched HCT. Our results show that signaling from GVHD target tissue PD-L1 interaction with PD-1 on donor T cells inhibits GSH/Myc pathways and causes dysfunctional metabolic reprogramming in activated STAT3-deficient but not Mito-STAT3–deficient donor T cells, leading to anergy, exhaustion, and apoptosis of the STAT3-deficient T cells, thereby preventing GVHD. At the same time, the lower expression of PD-L1 by host-type parenchymal cells in lymphohematopoietic tissues reduced PD-L1/PD-1 interactions and allowed functional metabolic reprogramming in the activated STAT3-deficient donor T cells despite reduction of GSH caused by STAT3 deficiency, leading to expansion of the T cells that mediate GVL activity. Therefore, both STAT3 deficiency and PD-1 signaling together are required to downregulate the GSH/Myc pathway and prevent tissue inflammation.

## Results

### STAT3-deficient donor T cells induce only mild and transient acute GVHD while preserving GVL activity.

**Figure 1:**
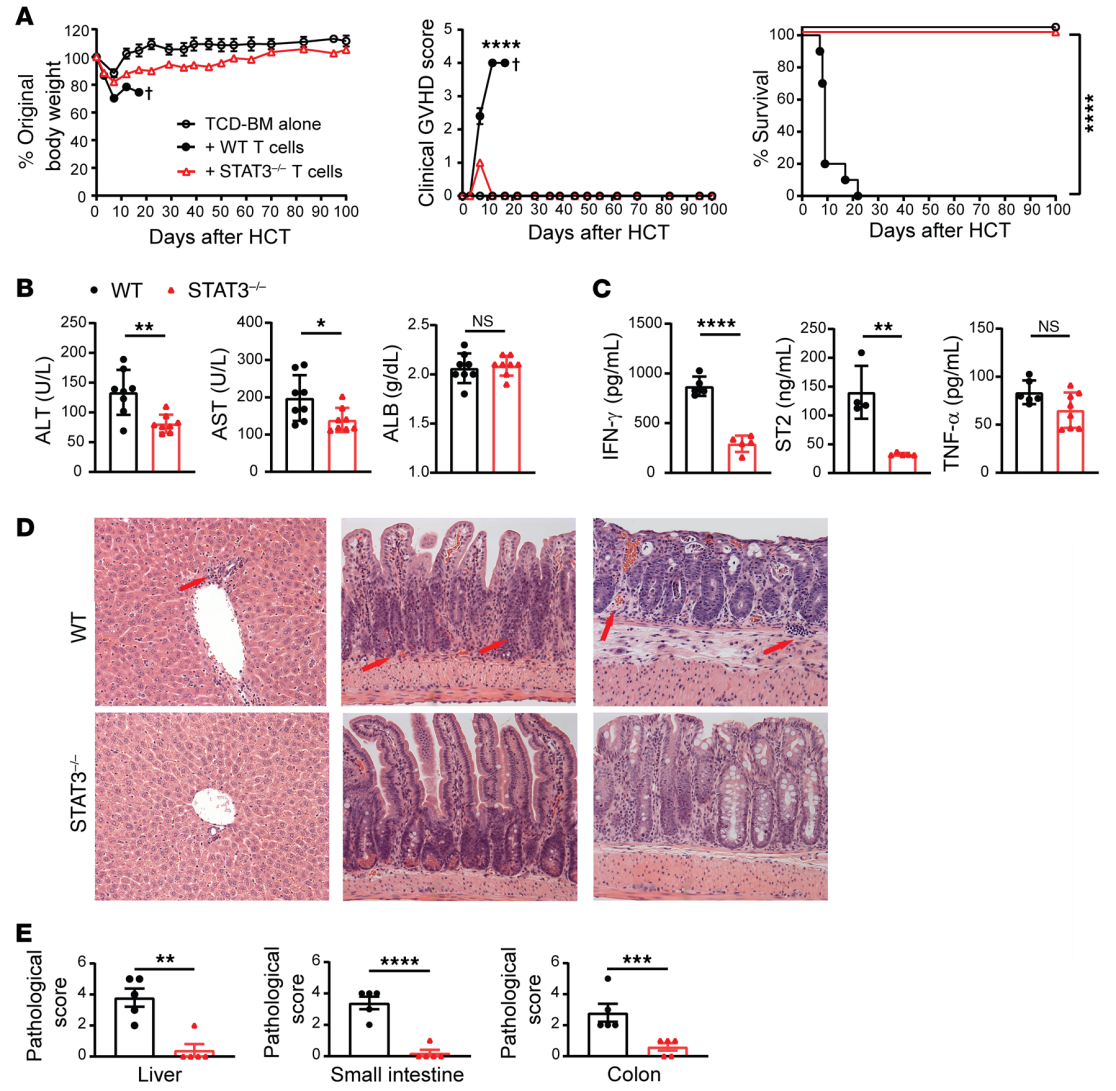
STAT3 deficiency in donor T cells prevents both acute and chronic GVHD. Lethally irradiated BALB/c recipients were engrafted with TCD-BM cells (5 × 10^6^) from WT C57BL/6 donors and CD90.2^+^ T cells (2.5 × 10^6^) from STAT3^–/–^ or WT C57BL/6 donors. (**A**) Curves of percentages of body weight change, clinical GVHD score, and percentage survival. *n* = 5 (TCD-BM); *n* = 10 (TCD-BM+WT T cells); *n* = 10 (TCD-BM+STAT3^–/–^ T cells) combined from 2 replicated experiments. (**B**) Serum concentrations of ALT, AST, and albumin (ALB) on day 6 are shown. *n* = 7–8 per group combined from 2 replicated experiments. (**C**) Serum concentration of IFN-γ, ST2, and TNF-α on day 6 after HCT are shown. *n* = 4–8 per group combined from 2 replicated experiments. (**D** and **E**) Histopathology of liver (left), small intestine (middle), and colon (right) evaluated on day 7 after HCT. Representative micrographic photos of liver, small intestine, and colon (**D**) and pathological scores of liver, small intestine, and colon are shown (**E**). Arrows point to infiltrating T cells or tissue damage area. Original magnification, ×200. *n* = 5 per group combined from 2 replicate experiments. Crosses indicate deaths. Data are represented as mean ± SEM. *P* values were calculated using nonlinear regression (curve fit) for body weight and clinical GVHD score comparisons (**A**), log-rank test for survival comparisons (**A**), and 2-tailed, unpaired Student’s *t* test for mean comparisons (**B**, **C**, and **E**). NS, *P* ≥ 0.05; *P < 0.05; **P < 0.01; ***P < 0.001, ****P < 0.0001.

STAT3 deficiency in donor T cells prevented both acute and chronic GVHD (12–14), but its mechanism remains unclear and its impact on GVL activity has not been evaluated. We first evaluated its impact on GVHD and GVL activity. Whole spleen cells (5 × 10^6^) or sorted Thy1.2^+^ T cells (2.5 × 10^6^) containing both CD4^+^ and CD8^+^ T cells from WT or STAT3^–/–^ C57BL/6 donors were coinjected with T cell–depleted bone marrow (TCD-BM) (5 × 10^6^) from WT C57BL/6 donors into lethally irradiated BALB/c recipients. The recipients were monitored for body weight changes, signs of clinical GVHD, and survival for up to 100 days after HCT. Whole spleen cells or sorted T cells from WT donors induced severe, fatal acute GVHD within 20 days after HCT. In contrast, STAT3^–/–^ spleen or T cells induced only mild and transient or reversible acute GVHD with no evidence of chronic GVHD, and all recipients (10/10) survived for more than 100 days (Figure 1A and Supplemental Figure 1A; supplemental material available online with this article; https://doi.org/10.1172/JCI165723DS1). GVHD prevention in recipients given STAT3^–/–^ T cells was evident with lower serum concentrations of alanine aminotransferase (ALT), aspartate aminotransferase (AST), soluble suppression of tumorigenicity 2 (sST2), and IFN-γ, although only slightly lower TNF-α concentrations (Figure 1, B and C) compared with that of recipients given WT T cells. The mild and transient acute GVHD was reflected by transient body weight loss and low clinical GVHD score at approximately 7 days after HCT (Figure 1A), but no obvious tissue damage was observed in recipients of STAT3^–/–^ donor T cells at 7 and 100 days after HCT (Figure 1, D and E, and Supplemental Figure 1, B and C). Thus, consistent with previous publications, STAT3^–/–^ T cells induce mild and transient or reversible acute GVHD with no evidence of chronic GVHD.

**Figure 2:**
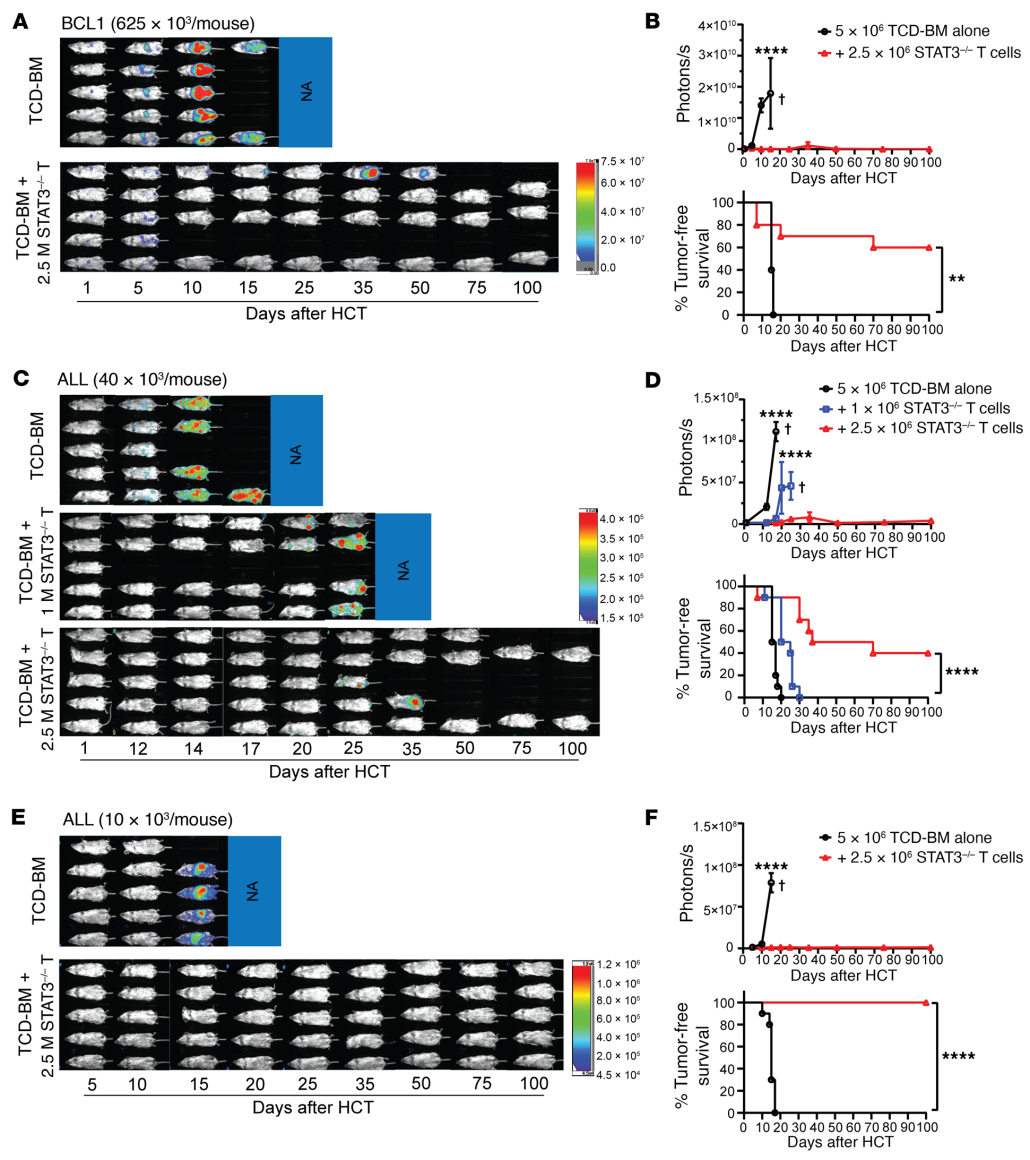
STAT3 deficiency in donor T cells preserves GVL activity. Lethally irradiated WT BALB/c recipients were engrafted with TCD-BM cells (5 × 10^6^) from WT C57BL/6 donors with or without CD90.2^+^ T cells (2.5 × 10^6^ [**A**–**F**] or 1 × 10^6^ [**C** and **D**]) from STAT3^–/–^ C57BL/6 donors on day 0. Recipients were then challenged with i.p. injection of BCL1/Luc cells (625 × 10^3^ [**A** and **B**] per mouse) or i.v. injection of ALL/Luc cells (40 × 10^3^ [**C** and **D**] or 10 × 10^3^ [**E** and **F**] per mouse) on day 0. (**A**, **C**, and **E**) Mice were monitored for tumor growth by using in vivo BLI after HCT. Representative BLI images of each mouse from each time point are shown. (**B**, **D**, and **F**) Curves of photon/second and percentage of tumor-free survival are shown. Data are represented as mean ± SEM. *n* = 9–10 per group combined from 2 replicated experiments. Crosses indicate deaths. *P* values were calculated using nonlinear regression (curve fit) for photon/second comparisons (**B**, **D**, and **F**) and log-rank test for survival comparisons (**B**, **D**, and **F**). **P < 0.01, ****P < 0.0001.

To test GVL activity of STAT3^–/–^ T cells, irradiated BALB/c mice were inoculated i.p. with luciferase-transfected BCL1 (BCL1/Luc^+^) leukemia/lymphoma cells (625 × 10^3^) and then engrafted with TCD-BM alone or TCD-BM plus STAT3^–/–^ Thy1.2^+^ T cells (2.5 × 10^6^). Recipients were monitored for BCL1 tumor growth with in vivo bioluminescent imaging (BLI) and were also monitored for survival. While all recipients (10/10) given TCD-BM alone died with progressive tumor growth by 20 days after HCT, addition of STAT3^–/–^ T cells eliminated the BCL1/Luc^+^ tumor cells by 10 days after HCT, and most (6/10) of the recipients survived for more than 100 days (Figure 2, A and B).

In further experiments, we tested GVL activity against acute lymphoblastic leukemia (ALL) cells (36), representing a disease with low susceptibility to GVL effects (37, 38). Irradiated BALB/c recipients were inoculated i.v. with ALL cells (40 × 10^3^/mouse) and then engrafted with 1.0 or 2.5 × 10^6^ STAT3^–/–^ T cells. With 2.5 × 10^6^ STAT3^–/–^ T cells added to the graft, the ALL cells were eliminated in approximately 50% of recipients, but all the recipients given 1.0 × 10^6^ STAT3^–/–^ T cells had progressive tumor growth (Figure 2, C and D). For unclear reasons, 2 recipients given 40 × 10^6^ ALL cells with STAT3^–/–^ donor T cells died without apparent tumor growth (Figure 2C). After inoculation of 10 × 10^3^ ALL cells, 2.5 × 10^6^ STAT3^–/–^ T cells were enough to prevent tumor growth in all (9/9) recipients, while all (9/9) recipients given TCD-BM alone had progressive tumor growth and died by 15 days after HCT (Figure 2, E and F). These results indicate that STAT3^–/–^ donor T cells can eliminate ALL tumor cells when the residual tumor cells are at low levels while preventing GVHD.

**Figure 3:**
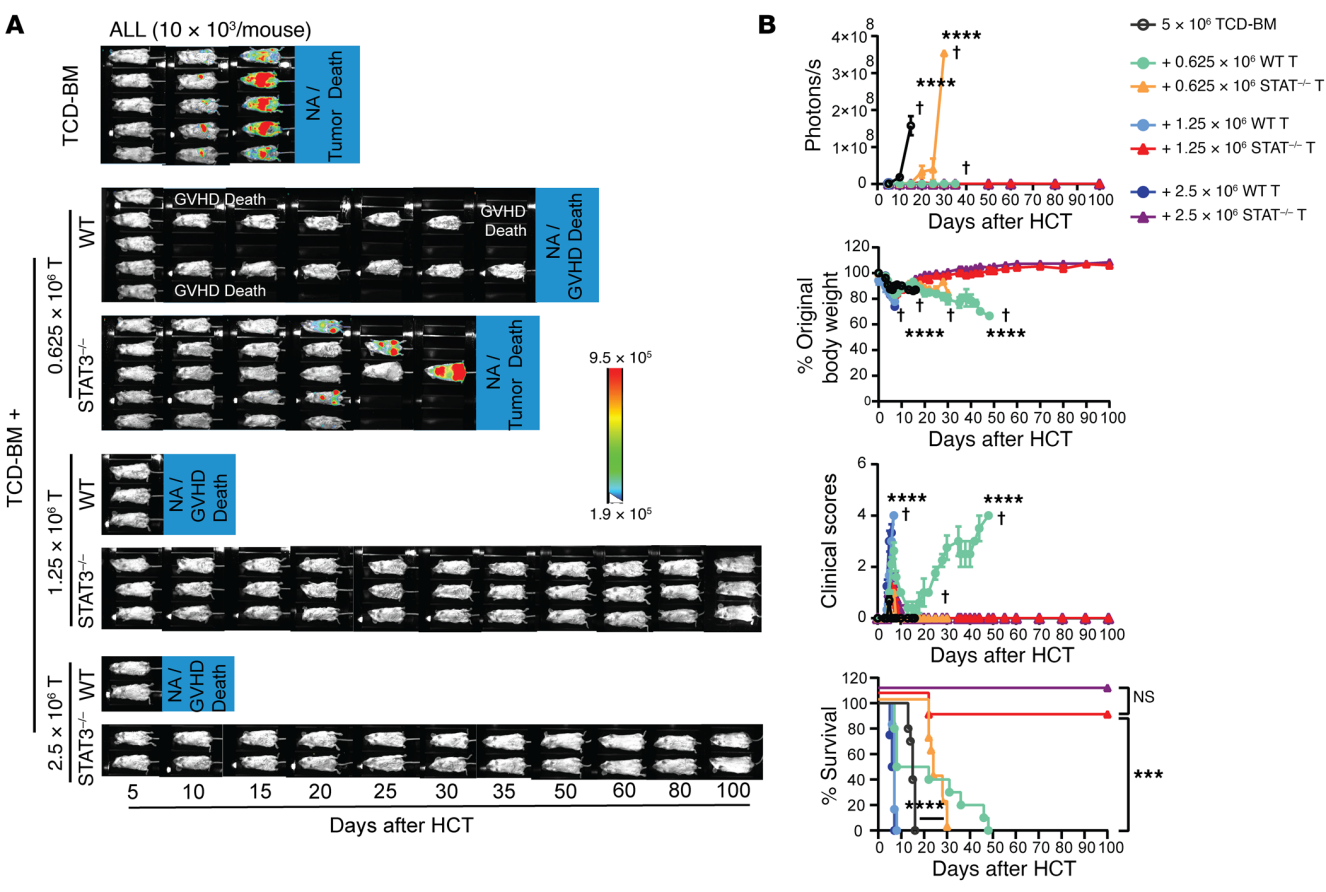
Comparison of GVL activity of WT and STAT3^–/–^ donor T cells. Lethally irradiated WT BALB/c recipients were i.v. inoculated with ALL/Luc cells (10 × 10^3^ per mouse) and engrafted with TCD-BM cells (5 × 10^6^) from WT C57BL/6 donors with or without CD90.2^+^ T cells (0.625, 1.25 or 2.5 × 10^6^) from WT or STAT3^–/–^ C57BL/6 donors on day 0. (**A**) Recipients were monitored for tumor growth by using in vivo BLI after HCT. Representative BLI images of each mouse from each time point are shown. (**B**) Curves of photon/second, percentages of original body weight, clinical GVHD score, and percentage survival are shown. Data are represented as mean ± SEM. *n* = 10 (TCD-BM); *n* = 10 (+0.625 × 10^6^ T); *n* = 6 (+1.25 × 10^6^ T); *n* = 4 (+2.5 × 10^6^ T) combined from 2 replicated experiments. Crosses indicate deaths. *P* values were calculated using nonlinear regression (curve fit) for photon/second comparisons (**B**) and log-rank test for survival comparisons (**B**). ***P < 0.001; ****P < 0.0001.

To further evaluate the GVL capacity of STAT3^–/–^ T cells in comparison with WT T cells, the recipients bearing 10 × 10^3^ ALL tumor cells were injected with titrated numbers (2.5 × 10^6^, 1.25 × 10^6^, 0.625 × 10^6^, and 0) of WT and STAT3^–/–^ T cells. Because we had observed that 2.5 × 10^6^ WT T cells induced lethal GVHD while the same numbers of STAT3^–/–^ T cells did not induce GVHD but preserved GVL activity (Figure 2E), we used graded numbers of recipients (2, 3, and 5) in each experiment to avoid unnecessary use of animals. This approach also allowed us to perform in vivo BLI for all recipients at the same time. Consistently, recipients without donor T cells all died of tumor growth within 20 days after HCT. All recipients given 2.5 to 0.625 × 10^6^ WT cells eliminated the ALL tumor cells, but the recipients given 2.5 or 1.25 × 10^6^ WT T cells died with acute GVHD within 10 days after HCT, and the recipients given 0.625 × 10^6^ WT T cells died with chronic GVHD within 50 days after HCT. In contrast, the recipients given 2.5 to 0.625 × 10^6^ STAT3^–/–^ T cells did not show obvious clinical GVHD, and nearly all recipients given 2.5 or 1.25 × 10^6^ STAT3^–/–^ T cells eliminated ALL tumor cells and survived for more than 100 days. A dose of 0.625 × 10^6^ STAT3^–/–^ T cells significantly prolonged survival, but did not eliminate ALL tumor cells (Figure 3, A and B). These results indicate that STAT3 deficiency in donor T cells prevents GVHD while preserving strong GVL activity that is mildly reduced compared with WT T cells. Taken together, these data show that STAT3^–/–^ T cells preserve strong GVL activity against not only BCL1 tumor cells but also non–GVL-susceptible ALL tumor cells, while inducing only mild and transient or reversible acute GVHD.

### Prevention of acute GVHD by STAT3 deficiency in donor T cells depends on target tissue PD-L1 interaction with PD-1 on infiltrating T cells.

Previous studies have suggested that STAT3 deficiency in donor T cells prevents GVHD by stabilizing natural Tregs (nTregs) and induced Tregs (iTregs) and augmenting thymic production of nTregs (12, 13). To determine whether other mechanisms might contribute to prevention of GVHD, we tested to ascertain whether in vivo depletion of Tregs alters the severity of GVHD induced by WT or STAT3^–/–^ T cells. Accordingly, deleting anti-CD25 mAbs (PC-61.5.3, 200 μg/mouse) (39) were injected i.p. on days 0, 2, 4, and 6 after HCT. Administration of anti-CD25 did not have any obvious effect on the severity of acute GVHD induced by WT or STAT3^–/–^ T cells (Supplemental Figure 2A), although it effectively depleted Foxp3^+^ Tregs in the spleen and GVHD target tissues such as liver (Supplemental Figure 2B). Therefore, factors unrelated to Tregs must explain how STAT3 deficiency in donor T cells prevents GVHD.

**Figure 4:**
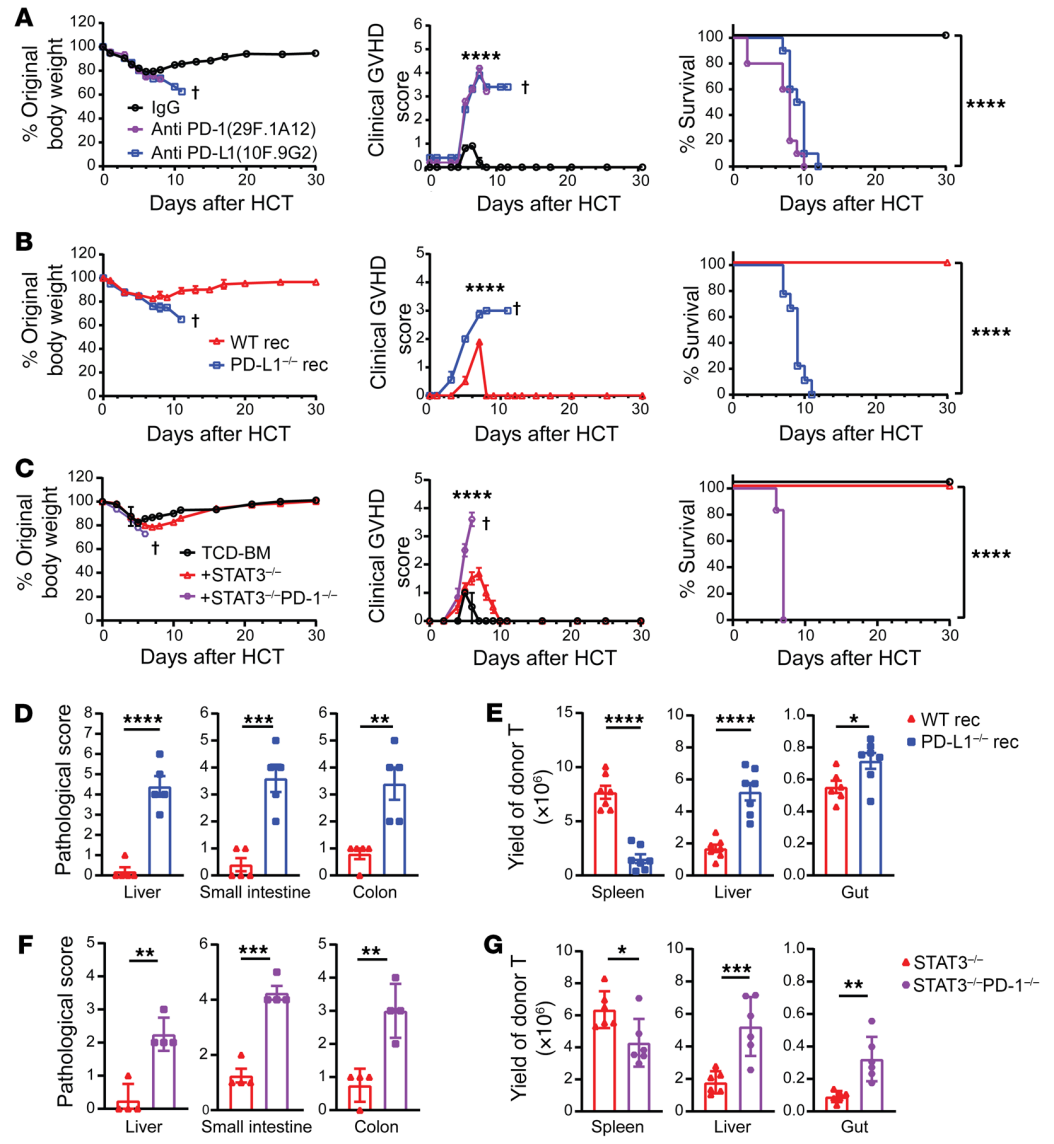
Prevention of acute GVHD by STAT3 deficiency in donor T cells requires PD-1 signaling triggered by GVHD target tissue expression of PD-L1. (**A**) Lethally irradiated BALB/c recipients were engrafted with TCD-BM cells (5 × 10^6^) and CD90.2^+^ T cells (2.5 × 10^6^) from STAT3^–/–^ C57BL/6 donors. Anti–PD-1 (29F.1A12; 200 μg/mouse) or anti–PD-L1 (10F.9G2; 200 μg/mouse) was given i.p. on days 4, 6, 8, and 10. Curves of percentages of original body weight, clinical GVHD score, and percentage survival are shown. (**B**, **D**, and **E**) Lethally irradiated WT or PD-L1^–/–^ BALB/c recipients were engrafted with TCD-BM cells (5 × 10^6^) from WT C57BL/6 donors and CD90.2^+^ T cells (2.5 × 10^6^) from STAT3^–/–^ C57BL/6 donors. (**B**) Curves of percentage original body weight, clinical GVHD score, and percentage survival are shown. (**C** and **F**–**G**) Lethally irradiated WT BALB/c recipients were engrafted with TCD-BM cells (5 × 10^6^) from WT C57BL/6 donors and CD90.2^+^ T cells (1 × 10^6^) from STAT3^–/–^ or STAT3^–/–^PD-1^–/–^ C57BL/6 donors. (**C**) Curves of percentage original body weight, clinical GVHD score, and percentage survival are shown. Data combined from 2 independent experiments. (**D** and **F**) Histopathology of liver, small intestine, and colon was evaluated on day 6 after HCT, and the histopathological scores are shown. *n* =4–5 combined from 2 replicate experiments. (**E** and **G**) Spleen, liver, small intestine, and colon samples were collected on day 6 after HCT, and yields of H-2Kb^+^TCR-β^+^ T cells are shown. *n* = 6–7 combined from 2 replicated experiments. Crosses indicate deaths. Data are represented as mean ± SEM. Two-tailed *P* values were calculated by nonlinear regression (curve fit) for comparison of body weights and clinical GVHD scores (**A**–**C**), log-rank test for survival comparisons (**A**–**C**), and unpaired Student’s *t* tests for other comparisons (**D**–**G**). *P < 0.05; **P < 0.01; ***P < 0.001; ****P < 0.0001.

Since host tissue PD-L1 reduced the severity of acute GVHD induced by donor CD4^+^ and CD8^+^ T cells together (10), we tested to determine whether prevention of acute GVHD by STAT3 deficiency in donor T cells depends on host-tissue expression of PD-L1. Accordingly, recipients of STAT3^–/–^ donor T cells were treated with anti–PD-L1 (10F. 9G2) or anti–PD-1 (29F.1A12) to block PD-L1/PD-1 interactions or control IgG on days 4, 6, 8, and 10 after HCT. Blockade of PD-L1/PD-1 interactions by anti–PD-L1 or anti–PD-1 resulted in lethal acute GVHD in recipients given STAT3^–/–^ donor T cells (Figure 4A). Consistently, STAT3^–/–^ donor T cells induced lethal acute GVHD in PD-L1^–/–^ recipients, but not in WT recipients (Figure 4B). In contrast, STAT3^–/–^PD-1^–/–^ donor T cells induced lethal acute GVHD in WT recipients (Figure 4C). The acute GVHD induced by STAT3^–/–^ donor T cells in PD-L1^–/–^ recipients (Figure 4, D and E) or by STAT3^–/–^PD-1^–/–^ donor T cells in WT recipients (Figure 4, F and G) caused severe damage in the liver, small intestine, and colon (Figure 4, D and F, and Supplemental Figure 3) with extensive infiltration of donor T cells in those tissues, but not in the spleen (Figure 4, E and G). These results indicate that prevention of acute GVHD by STAT3 deficiency in donor T cells depends on host-tissue PD-L1 interaction with PD-1 on infiltrating donor T cells.

### STAT3 deficiency in donor T cells augments tissue-specific deletion of host-reactive T cell clones and maintains tolerance of the residual T clones in GVHD target tissues in a PD-L1/PD-1 interaction–dependent manner.

**Figure 5:**
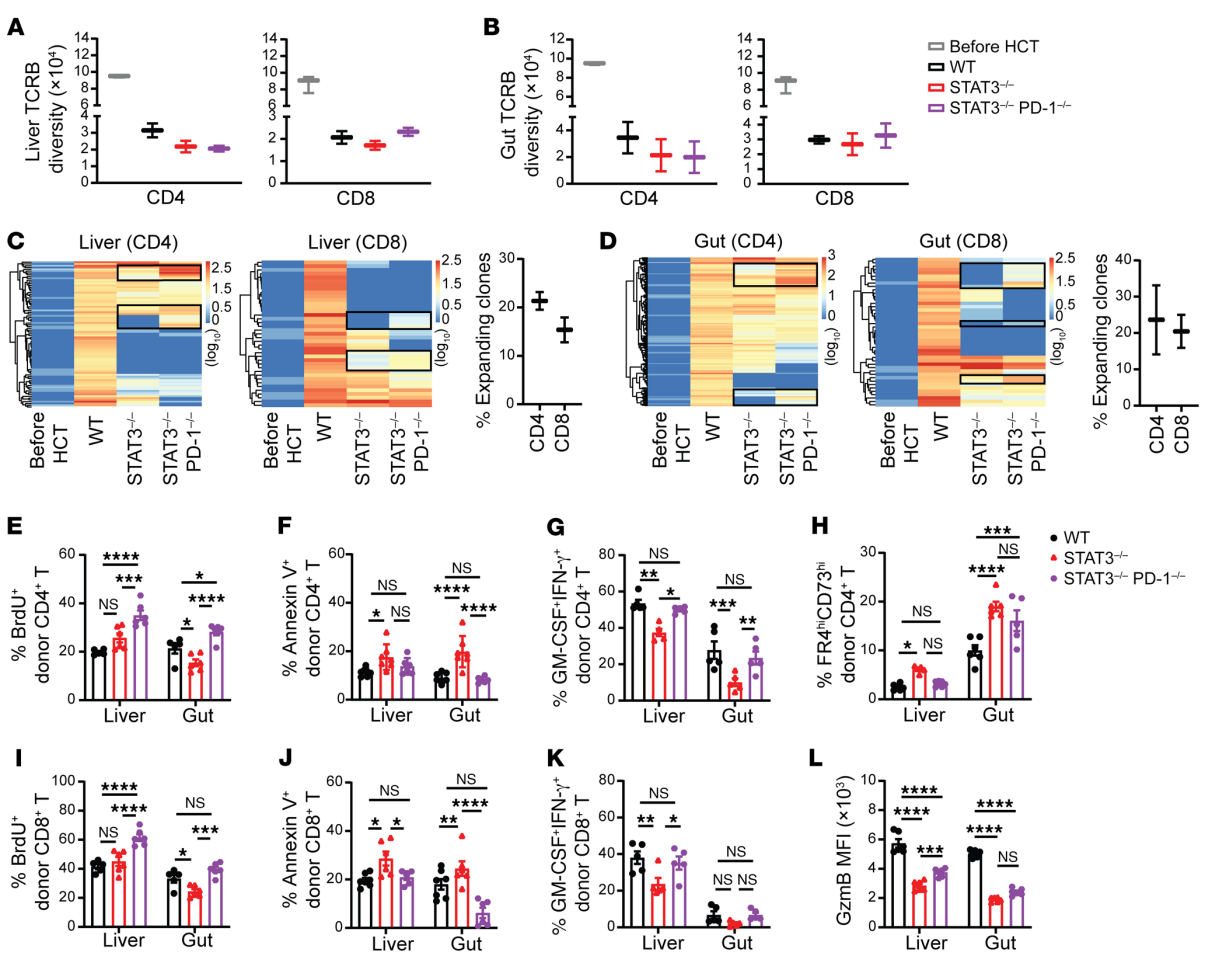
STAT3 deficiency in donor T cells augments anergy, exhaustion, and apoptosis of infiltrating T cells in GVHD target tissues in a PD-L1/PD-1 signaling–dependent manner. Lethally irradiated BALB/c recipients were engrafted with TCD-BM cells (5 × 10^6^) from WT C57BL/6 donors and CD90.2^+^ T cells (1 × 10^6^) from WT or STAT3^–/–^ or STAT3^–/–^PD-1^–/–^ C57BL/6 donors. (**A** and **B**) FACS-sorted donor-type CD4^+^ and CD8^+^ T cells from liver and gut were analyzed with RNA-Seq and TCR-CDR3-Seq microarray. (**A**) TCR-CDR3 diversity of TCRB of CD4^+^ and CD8^+^ T in the liver were compared. (**B**) TCR-CDR3 diversity of TCRB of CD4^+^ and CD8^+^ T in gut the were compared. (**C**) Heatmaps of host-reactive TCRB in liver were compared. (**D**) Heatmaps of host-reactive TCRB in gut were compared. TCR-CDR3-Seq measurements were performed on duplicate samples from each group. The numbers are log-transformed (base 10 with offset of 1) TCR frequency, which have been normalized to counts per million. Each sample contained lymphocytes from 3 recipients. (**E**–**L**) On day 6 after HCT, CD4^+^ and CD8^+^ T cells from the liver and gut of recipients in each group were analyzed with flow cytometry. (**E** and **I**) Percentage BrdU^+^. *n* = 5–6 per group combined from 2 replicated experiments. (**F** and **J**) Percentage Annexin-V^+^. *n* = 6–7 per group combined from 2 replicated experiments. (**G** and **K**) Percentage GM-CSF^+^IFN-γ^+^. *n* = 5 per group combined from 2 replicated experiments. (**H**) Percentage FR4^hi^CD73^hi^ among CD4^+^ T cells and (**L**) MFI of GzmB of CD8^+^T are shown. *n* = 5–6 per group combined from 2 replicated experiments. Data are represented as mean ± SEM (**A**, **B**, **E**–**L**). *P* values were calculated using 2-way ANOVA (**E**–**L**). NS, *P* ≥ 0.05; *P < 0.05; **P < 0.01; ***P < 0.001; ****P < 0.0001.

We evaluated the extent to which the TCR repertoire of donor T cells infiltrating GVHD target tissues was affected by STAT3 deficiency. Using TCR-CDR3-Seq analysis (40, 41) , we measured TCR repertoires of donor CD4^+^ and CD8^+^ T cells before HCT and in GVHD target tissues (liver and gut) at 6 days after HCT. The TCR-CDR3 diversity of T cells from WT, STAT3^–/–^, or STAT3^–/–^PD-1^–/–^ donors before HCT were similar and highly diverse, and the data were combined into a single group of “T cells before HCT” (Figure 5, A and B, and Supplemental Figure 4, A and B). As compared with T cells before HCT, donor CD4^+^ and CD8^+^ T cells from the liver and gut tissues of GVHD recipients given WT T cells showed much lower TCRB and TCRA diversity, reflecting the expansion of alloreactive clones (Figure 5, A and B, and Supplemental Figure 4, A and B). Donor CD4^+^ and CD8^+^ T cells from the liver and gut tissues of GVHD recipients given STAT3^–/–^ T cells or STAT3^–/–^PD-1^–/–^ T cells showed similarly low TCRB and TCRA diversity and no statistically significant differences as compared with CD4^+^ and CD8^+^ T cells from recipients given WT donor T cells (Figure 5, A and B, and Supplemental Figure 4, A and B). These results indicate that, consistent with previous reports (42, 43), acute GVHD is associated with reduction of donor T cell diversity. However, prevention of acute GVHD by STAT3 deficiency in donor T cells did not prevent reduction of donor T cell diversity, and induction of acute GVHD by STAT3^–/–^PD-1^–/–^ T cells did not further reduce donor T cell diversity.

In addition, we used TCRB-Seq and TCRA-Seq to identify host-reactive clonotypes with frequencies of less than 10^–5^ before HCT and more than 10^–4^ among donor T cells from GVHD target tissues of recipients on day 6 after HCT. The host-reactive CD4^+^ and CD8^+^ T cell clonotypes in the liver and gut of recipients given WT donor T cells were markedly expanded. Many of these same host-reactive CD4^+^ and CD8^+^ T cell clonotypes were not expanded in the liver and gut of recipients given STAT3^–/–^ or STAT3^–/–^PD-1^–/–^ donor T cells (Figure 5, C and D, and Supplemental Figure 4, C and D). Although the overall clonotype expansions were similar for STAT3^–/–^ donor T cells that did not cause GVHD and STAT3^–/–^PD-1^–/–^ T cells that did cause GVHD, approximately 15% to 30% of TCRB and TCRA clonotypes that were not expanded among CD4^+^ or CD8^+^ T cells from STAT3^–/–^ donors were expanded among CD4^+^ or CD8^+^ T cells from STAT3^–/–^PD-1^–/–^ donors (Figure 5, C and D, and Supplemental Figure 4, C and D). Most (~90%) of the expanded TCRA and TCRB clonotypes represented in donor T CD4^+^ and CD8^+^ cells differed between the liver and gut with very little overlapping (Supplemental Figure 4E).

We further analyzed the tolerance status of residual donor CD4^+^ and CD8^+^ T cells in the GVHD target tissues on day 6 after HCT. As compared with WT CD4^+^ T cells, STAT3^–/–^ CD4^+^ T cells in the liver and gut had similar or lower proliferation rates, as indicated by BrdU^+^ staining, but higher apoptosis rates, as indicated by annexin V^+^ staining, lower pathogenic activity, as indicated by the lower frequency of cells with a GM-CSF^+^IFN-γ^+^ cytokine profile, and higher frequencies of anergic cells, as indicated by FR4^hi^CD73^hi^ staining (44–46) (Figure 5, E–H, and Supplemental Figure 5). The results with STAT3^–/–^PD-1^–/–^CD4^+^ T cells indicated that these differences depend on PD-L1/PD-1 signaling. As compared with WT CD8^+^ T cells, STAT3^–/–^CD8^+^ T cells in the liver and gut also had similar or lower proliferation, increased apoptosis, lower pathogenic cytokine production, and lower granzyme B expression (Figure 5, I–L, and Supplemental Figure 6). The results with STAT3^–/–^PD-1^–/–^ CD8^+^ T cells also indicated that, with the exception of pathogenic cytokine production and granzyme B in the gut, these differences depend on PD-L1/PD-1 signaling (Figure 5, I–L, and Supplemental Figure 6). Taken together, these results indicate that STAT3-deficient donor T cells exhibit tissue-specific clonal expansion in the liver and gut, but PD-L1/PD-1 interactions limit their proliferation, induce apoptosis, or impede effector functions, thereby preventing their ability to cause GVHD.

### STAT3 deficiency in donor T cells augments host-tissue PD-L1/PD-1 signaling–mediated inhibition of the GSH/Myc pathway, leading to metabolic dysfunction of T cells that infiltrate GVHD target tissues.

**Figure 6:**
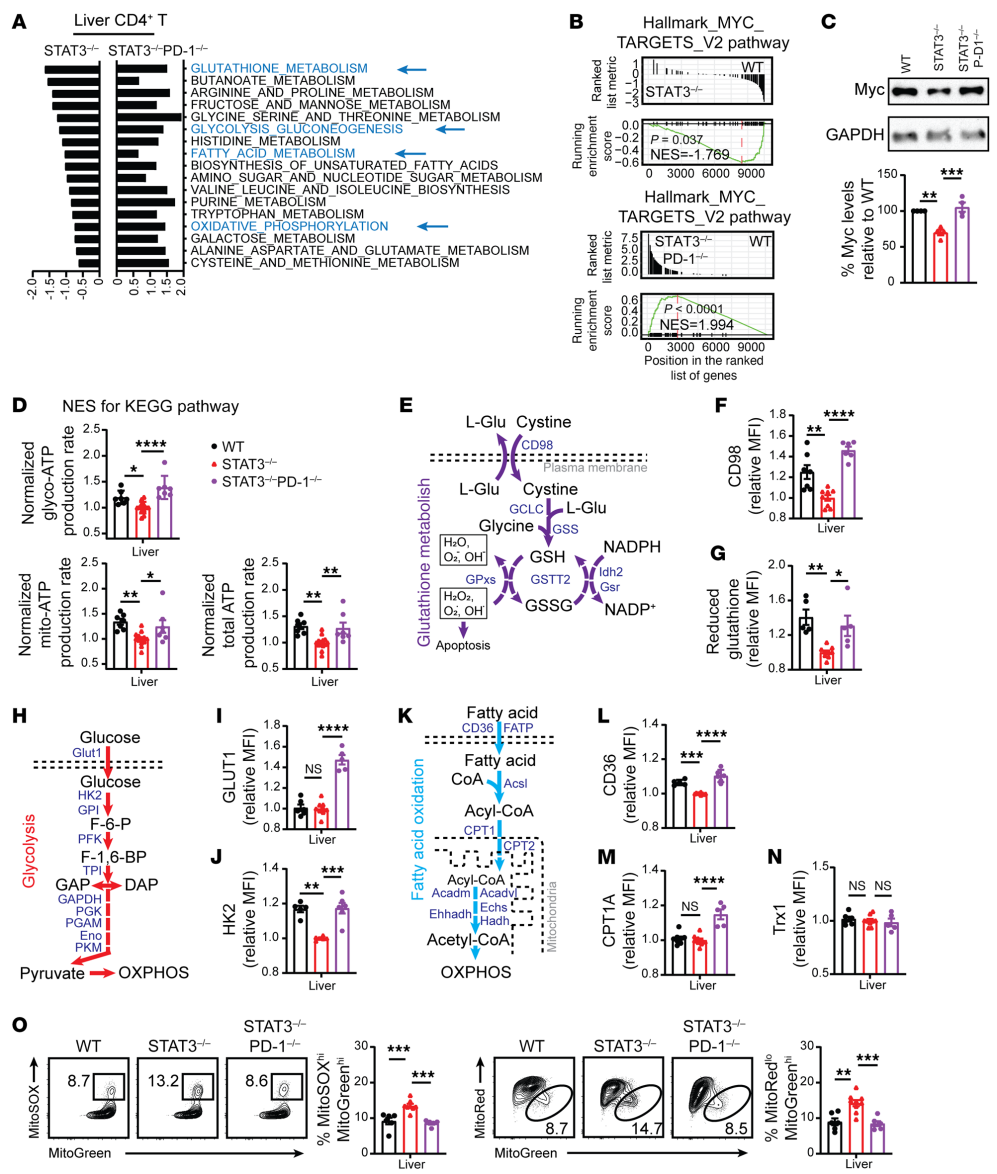
Stat3 deficiency in donor T cells augments PD-1–mediated inhibition of GSH/Myc pathways and production of Mito-ROS. Lethally irradiated BALB/c recipients were engrafted with TCD-BM and CD90.2^+^ T cells from WT, STAT3^–/–^, or STAT3^–/–^PD-1^–/–^ C57BL/6 donors as described for Figure 5. On day 6 after HCT, CD90.2^+^ T cells from liver were isolated for RNA-Seq, Seahorse, immunoblotting, and flow-cytometry analysis. Data are combined from at least 2 replicate experiments. (**A**) NES of KEGG pathway activity of CD4^+^ T cells, setting the activity of WT CD4^+^ T cells as the reference, for comparisons with STAT3^–/–^ and STAT3^–/–^PD-1^–/–^ T cells. (**B**) GSEA plots of MYC target V2 pathway-related gene set expression in WT, STAT3^–/–^, and STAT3^–/–^PD-1^–/–^ donor CD4^+^ T cells. (**C**) Myc protein was measured by immunoblotting. (**D**) Glyco-ATP, Mito-ATP, and total ATP. *n* = 7–14. (**E**) The GSH metabolism pathway is shown. (**F** and **G**) MFI of CD98 and reduced GSH of CD4^+^ T cells in the liver. *n* = 5–9. (**H**) Glycolysis pathway is shown. (**I** and **J**) MFI of GLUT1 and HK2 of CD4^+^ T cells. *n* = 5–9. (**K**) FAO pathway is shown. (**L** and **M**) MFI of CD36 and CPT1A of CD4^+^ T cells. *n* = 5–9. (**N**) MFI of Trx1 of CD4^+^ T cells. *n* = 5–9. (**O**) Representative flow cytometry pattern and mean ± SEM of percentage MitoSOX^hi^MitoGreen^hi^ and percentage MitoRed^lo^MitoGreen^hi^ CD4^+^ T cells. *n* = 5–9. Separate experiments were performed with WT versus STAT3^–/–^ or STAT3^–/–^ versus STAT3^–/–^PD-1^–/–^ in **D**, **F**, **G**, **I**, **J**, **L**, **M**, **N**, and results were normalized to the mean values for STAT-3–deficient cells. Data are represented as mean ± SEM. *P* values were calculated by using 1-way ANOVA (**C**, **D**, **F**, **G**, **I**, **J**, **L**, and **O**). NS, *P* ≥ 0.05; *P < 0.05; **P < 0.01; ***P < 0.001; ****P < 0.0001.

Antioxidative GSH plays an important role in metabolic integration and reprogramming by scavenging ROS and increasing MYC pathway activity during inflammatory T cell responses (35). Therefore, we tested to determine whether PD-1 signaling alters GSH regulation of metabolic reprogramming in STAT3-deficient donor T cells from GVHD target tissues. Using RNA-Seq analysis, we compared the metabolic programming pathways of CD4^+^ and CD8^+^ T cells from the liver tissues of recipients given WT, STAT3^–/–^, or STAT3^–/–^PD-1^–/–^ T cells at 6 days after HCT. As compared with WT CD4^+^ T cells, GSH metabolism, glycolysis-gluconeogenesis, fatty acid metabolism, and oxidative phosphorylation pathways appeared to lower activity in STAT3^–/–^ CD4^+^ T cells but to increase activity in STAT3^–/–^PD-1^–/–^ CD4^+^ T cells (Figure 6A). The pattern was similar for GSH metabolism and oxidative phosphorylation in STAT3^–/–^ and STAT3^–/–^PD-1^–/–^ CD8^+^ T cells (Supplemental Figure 7A). Although STAT3^–/–^CD8^+^ T cells did not show reduction in fatty acid metabolism and oxidative phosphorylation pathways, those were still increased in STAT3^–/–^PD-1^–/–^ CD8^+^ T cells (Supplemental Figure 7A). Myc-dependent metabolic pathway activity was lower in STAT3^–/–^ CD4^+^ and CD8^+^ T cells than in WT T cells, but markedly higher in STAT3^–/–^PD-1^–/–^ CD4^+^ and CD8^+^ T cells, as indicated by normalized enrichment score (NES) (Figure 6B and Supplemental Figure 7B). Myc protein expression was also lower in Thy1.2^+^ STAT3^–/–^ T cells than in WT T cells, but was unaffected in STAT3^–/–^PD-1^–/–^ T cells (Figure 6C).

With the Seahorse XF Real-Time ATP Rate Assay, which can simultaneously measure oxygen consumption rate (OCR) (Mito-ATP) and extracellular acidification rate (ECAR) (Glyco-ATP) (47), we compared Mito-ATP and Glyco-ATP production by WT versus STAT3^–/–^ T cells and by STAT3^–/–^ versus STAT3^–/–^PD-1^–/–^ T cells (Supplemental Figure 8). Mito-ATP, Glyco-ATP, and total ATP production were lower in STAT3^–/–^ total T cells than in WT cells, but were unaffected in STAT3^–/–^PD-1^–/–^ T cells (Figure 6D). These results suggest that STAT3 deficiency in donor T cells may augment PD-1–mediated inhibition of GSH synthesis, thereby causing metabolic dysfunction.

We dissected how STAT3 deficiency in donor T cells inhibits GSH synthesis. As described in the diagram, GSH synthesis requires efflux of l-glutamine and influx of cystine through cell membrane glutamine transporter CD98 as well as the rate-limiting enzyme GCLC (48) (Figure 6E). Cell-surface expression of CD98 was lower in STAT3^–/–^ CD4^+^ and CD8^+^ T cells than in WT T cells, but was unaffected or higher in STAT3^–/–^PD-1^–/–^ T cells (Figure 6F, Supplemental Figure 9A, and Supplemental Figure 10A). GSH measured by the MFI of Thiol Green staining was lower in STAT3^–/–^ CD4^+^ and CD8^+^ T cells than in WT T cells, but was unaffected in STAT3^–/–^PD-1^–/–^ T cells (Figure 6G, Supplemental Figure 9B, and Supplemental Figure 10B). In previous studies, an in vitro cross-linking assay showed that PD-1 signaling reduced GSH synthesis in activated CD4^+^ T cells (49). Thus, our results indicate that STAT3 deficiency in donor T cells augments inhibition of GSH synthesis mediated by PD-1 signaling.

Reduction of GSH synthesis by GCLC deficiency decreased TCR-driven Myc upregulation and reduced glycolysis and fatty acid oxidation (FAO) (35, 50). Glycolysis is regulated by the membrane glucose transporter Glut 1 and the rate-limiting enzyme HK2 and other related enzymes (51, 52) (Figure 6H). Expression of Glut1 protein was not affected by STAT3 deficiency, but was higher in STAT3^–/–^PD-1^–/–^ CD4^+^ and CD8^+^ T cells than in WT T cells (Figure 6I, Supplemental Figure 9C, and Supplemental Figure 10C). Expression of HK2 protein was lower in STAT3^–/–^ CD4^+^ and CD8^+^ T cells than in WT T cells, but was unaffected in STAT3^–/–^PD-1^–/–^ T cells (Figure 6J, Supplemental Figure 9D, and Supplemental Figure 10D). In a previous study, PD-1 signaling downregulated T cell Glut1 and HK2 protein expression (49). Thus, our results indicate that STAT3 deficiency in donor T cells augments inhibition of HK2 expression mediated by PD-1 signaling.

FAO is regulated by the membrane fatty acid transporter CD36 and the mitochondrial membrane rate-limiting enzyme CPT1a (53) (Figure 6K). Protein expression of CD36 was lower in STAT3^–/–^ donor CD4^+^ and CD8^+^ T cells than in WT T cells, but was not affected in STAT3^–/–^PD-1^–/–^ T cells (Figure 6L, Supplemental Figure 9E, and Supplemental Figure 10E). Expression of CPT1A was not affected by STAT3 deficiency, but was higher in STAT3^–/–^PD-1^–/–^ CD4^+^ and CD8^+^ T cells than in WT T cells (Figure 6M, Supplemental Figure 9F, and Supplemental Figure 10F). These results suggest that STAT3 deficiency in donor T cells augments PD-1 signaling–mediated inhibition of CD36.

Mitochondria produce ROS (Mito-ROS), and its production is increased by PD-1 signaling, but decreased by mitochondrial STAT3 (Mito-STAT3) (19, 23). Although low concentrations of ROS act as signaling messengers and modify protein function or structure by oxidation, high concentrations of ROS lead to cell death (54, 55). GSH and thioredoxin-1 (Trx1) in the cytosol play critical roles in buffering ROS to allow metabolic reprogramming during T cell activation (30, 35). Expression of GSH was lower in STAT3^–/–^ CD4^+^ and CD8^+^ T cells than in WT T cells, but was not affected in STAT3^–/–^PD-1^–/–^ T cells (Figure 6G and Supplemental Figure 10B). TRX1 expression did not differ among WT, STAT3^–/–^, and STAT3^–/–^PD-1^–/–^ CD4^+^ and CD8^+^ T cells (Figure 6N and Supplemental Figure 10G). Mito-ROS production, as indicated by MitoSOX^hi^MitoGreen^hi^ staining, and Mito-dysfunction, as indicated by MitoRed^lo^MitoGreen^hi^ staining, were higher in STAT3^–/–^ CD4^+^ and CD8^+^ T cells than in WT T cells, but were not affected in STAT3^–/–^PD-1^–/–^ T cells (Figure 6O and Supplemental Figure 10H). These results indicate that STAT3 deficiency and PD-1 signaling in donor T cells synergistically augment Mito-ROS production and reduce GSH synthesis, increasing Mito-dysfunction that can augment T cell anergy and apoptosis.

**Figure 7:**
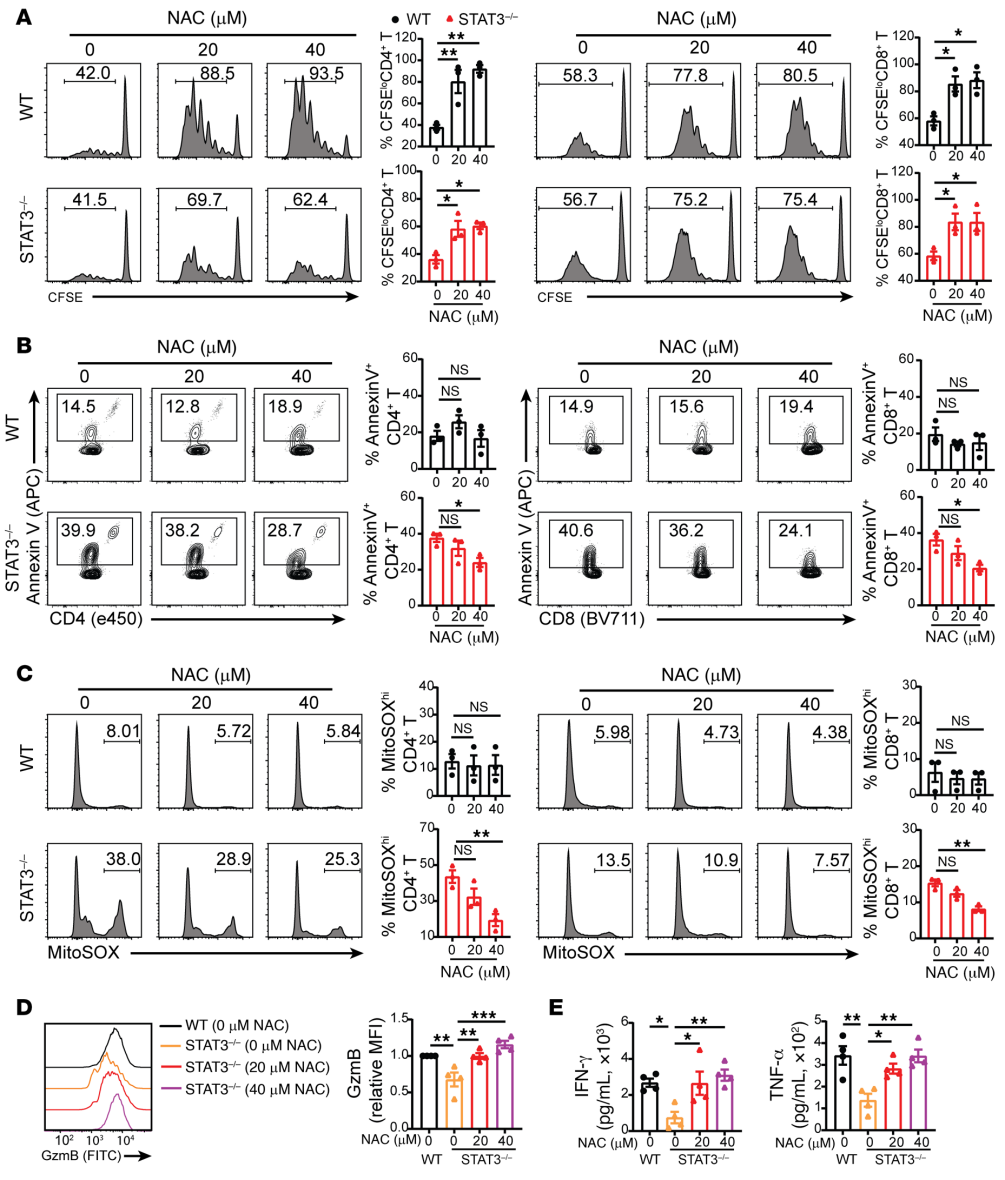
Antioxidant NAC rescues allogeneic donor STAT3^–/–^ T cell function in vitro. CSFE-labeled WT or STAT3^–/–^ CD90.2^+^ T cells from C57BL/6 were cocultured with irradiated BALB/c DCs. The cells were treated with antioxidant NAC at 0, 20, or 40 μM on day 0. (**A**) T cells were collected on day 4 after coculture for flow cytometric analysis of CD8^+^ and CD4^+^ T cells, respectively. Representative flow cytometry pattern and mean ± SEM of percentages of CFSE^lo^H-2Kb^+^TCR-β^+^CD4^+^ and CD8^+^ cells are shown. *n* = 3 per group combined from 3 replicated experiments. (**B**–**D**) Cells or supernatant was collected on day 4 for analysis. (**B**) Representative flow cytometry pattern and mean ± SEM of percentage of AnnexinV^+^CD4^+^ and CD8^+^ T cells are shown. *n* = 3 per group combined from 3 replicated experiments. (**C**) Representative flow cytometry pattern and mean ± SEM of percentage of MitoSOX^hi^CD4^+^ and CD8^+^ T cells are shown. *n* = 3 per group combined from 3 replicated experiments. (**D**) Representative flow cytometry pattern and mean ± SEM of MFI of GzmB are shown. *n* = 4 per group combined from 4 replicated experiments. (**E**) Concentrations of INF-γ and TNF-α in supernatants. *n* = 4 per group combined from 4 replicated experiments. Data are represented as mean ± SEM. *P* values were calculated using 1-way ANOVA. NS, *P* ≥ 0.05; *P < 0.05; **P < 0.01; ***P < 0.001; ****P < 0.0001.

To validate the role of ROS in augmenting apoptosis of STAT3^–/–^ donor T cells, we tested to determine whether the antioxidant reagent *N*-acetyl cysteine (NAC) could rescue the function of STAT3^–/–^ T cells during stimulation in mixed lymphocyte reaction (MLR) in vitro. NAC increased the proliferation of both WT and STAT3^–/–^ T cells. At the 20 and 40 μM concentrations tested, NAC decreased apoptosis of STAT3^–/–^, but not WT, T cells and decreased mitochondrial dysfunction in STAT3^–/–^, but not in WT, T cells (Figure 7, A–C). NAC also increased granzyme B expression in STAT3^–/–^ CD8^+^ T cells (Figure 7D) and increased IFN-γ and TNF-α concentrations in the culture supernatant of STAT3^–/–^ CD4^+^ and CD8^+^ T cells (Figure 7E). When STAT3^–/–^ T cells were stimulated with PD-L1^–/–^ instead of WT host-type DCs in the MLR, the effects of NAC disappeared (Supplemental Figure 11), showing that they depend on PD-L1/PD-1 signaling. However, administration of antioxidant reagents NAC or MnTBAP had no clearly demonstrable effects, suggesting enhanced function of STAT3^–/–^ T cells to cause GVHD in vivo (Supplemental Figure 12), although previous studies showed that antioxidants augmented acute GVHD induced by WT T cells (20).

### STAT3^–/–^ T cells in lymphohematopoietic tissues show no significant reduction in GSH/Myc pathway activities, despite reduced expression of GSH.

**Figure 8:**
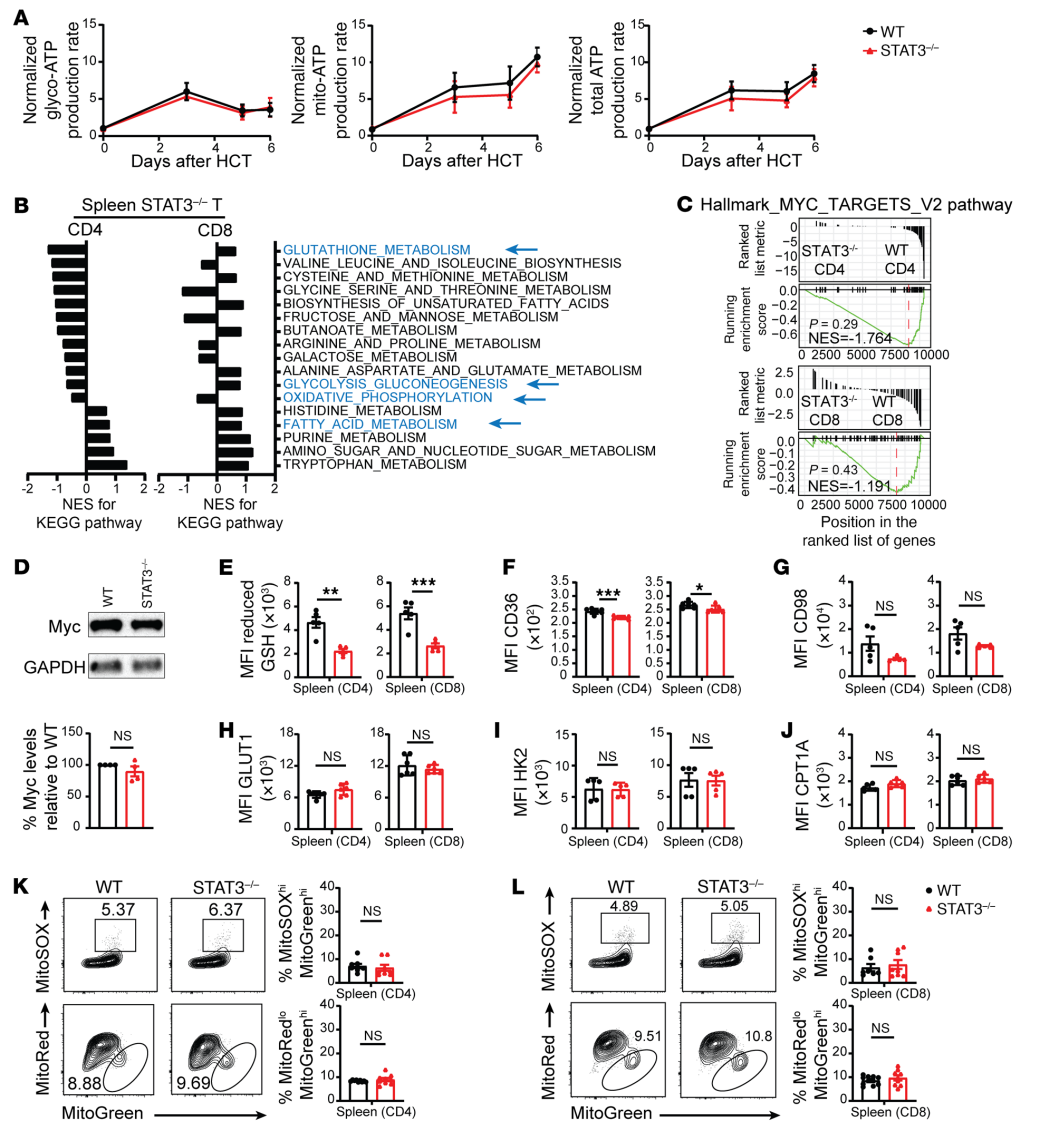
Splenic Stat3^–/–^ donor T cells do not have significant inhibition of GSH/Myc pathway activity. Lethally irradiated BALB/c recipients were engrafted with TCD-BM (5 × 10^6^) from WT C57BL/6 donors and CD90.2^+^ T cells (1 × 10^6^) from WT or STAT3^–/–^ C57BL/6 donors. (**A**) On days 0, 3, 5, and 6 after HCT, CD90.2^+^ T cells from spleen were isolated for Seahorse analysis. Normalized Glyco-ATP, Mito-ATP, and total ATP production rates are shown, using mean STAT3^–/–^ values as the reference. *n* = 2 per group combined from 2 replicates; each sample contained lymphocytes from 3 recipients. (**B**–**L**) On day 6 after HCT, lymphocytes from spleen were isolated for RNA-Seq, immunoblotting, and flow cytometry analysis. (**B**) NES of KEGG pathway activity of CD4^+^ and CD8^+^T cells are shown, setting the WT as the reference. (**C**) MYC target V2 pathway–related gene set expression in CD4^+^ (top) and CD8^+^ (bottom) T cells were compared between WT and STAT3^–/–^ donors. GSEA plots are shown. (**D**) Myc protein in Thy1.2^+^ T cells was measured by immunoblotting. (**E**–**J**) Means ± SEM of MFI of CD98 reduced GSH (**E**), CD36 (**F**), CD98 (**G**), GLUT1 (**H**), HK2 (**I**), and CPT1A (**J**) of CD4^+^ and CD8^+^ T cells in the spleen. *n* = 5–7 per group combined from 2 replicates. (**K** and **L**) Representative flow cytometry patterns and means ± SEM of percentages of MitoSOX^hi^MitoGreen^hi^ and percentages of MitoRed^lo^MitoGreen^hi^ of CD4^+^ (**K**) and CD8^+^ T cells (**L**) from the spleen of different groups are compared, *n* = 8–9 per group combined from 3 replicates. *P* values were calculated by unpaired Student’s *t* tests. NS, *P* ≥ 0.05; *P < 0.05; **P < 0.01; ***P < 0.001.

We compared the metabolic profiles of splenic T cells from GVHD recipients given WT versus non-GVHD recipients given STAT3^–/–^ donor T cells. We first measured kinetics of glycolysis and OXPHOS in WT and STAT3^–/–^ donor splenic T cells at days 0, 3, 5, and 6 after HCT (Figure 7A). The WT and STAT3^–/–^ T cells had similar rates of Glyco- and Mito-ATP production before HCT, followed by a burst of Glyco- and Mito-ATP production in both WT and STAT3^–/–^ donor T cells after HCT, reaching peaks on day 3 and day 6, respectively (Figure 8A). Glyco-ATP production declined between days 3 and 5, but Mito-ATP production appeared to be sustained until day 5 and then increased afterwards (Figure 8A). STAT3 deficiency had no effect on Glyco-ATP production and caused only a slight reduction in Mito-ATP production (Figure 8A). RNA-Seq analysis on day 6 after HCT suggested that STAT3 deficiency had less effect in reducing GSH metabolism, glycolysis-gluconeogenesis, and oxidative phosphorylation in CD4^+^ T cells from the spleen than in those from the liver (Supplemental Figure 13A). In addition, STAT3 deficiency increased fatty acid metabolism in splenic CD4^+^ T cells, but decreased fatty acid metabolism in hepatic CD4^+^ T cells (Supplemental Figure 13A). STAT3 deficiency increased GSH metabolism in splenic CD8^+^ T cells, but decreased GSH metabolism in hepatic CD8^+^ T cells (Supplemental Figure 13B). These differences reflect the lower PD-L1/PD-1 signaling to the donor T cells in the spleen than in the liver. Consistent with our previous report that the ratio of PD-L1–expressing host-type parenchymal cells versus lymphoid cells in the gut was more than 20-fold higher than that in the spleen early after HCT (15), we also observed that the ratio of PD-L1–expressing host-type parenchymal cells in the liver was approximately 20-fold higher than that in the spleen; in addition, the higher levels of expression of PD-L1 by hepatocytes were validated by immunofluorescent staining (Supplemental Figure 14).

RNA-Seq analysis also showed differences in the effects of STAT3 deficiency between splenic CD4^+^ T cells and CD8^+^ T cells. STAT3 deficiency decreased GSH metabolism and glycolysis-gluconeogenesis in splenic CD4^+^ T cells, but increased these pathways in splenic CD8^+^ T cells (Figure 8B). Although the NES appeared to be reduced in the STAT3^–/–^ T cells as compared with WT T cells, STAT3 deficiency did not significantly decrease Myc-dependent metabolic pathway activity in splenic CD4^+^ or CD8^+^ T cells and did not affect Myc protein expression in Thy1.2^+^ T cells (Figure 8, C and D), unlike STAT3-deficient T cells in the liver (Figure 6, B and C). Although GSH measured by the MFI of Thiol Green staining and CD36 was significantly lower in splenic STAT3^–/–^ CD4^+^ and CD8^+^ T cells than in WT T cells (Figure 8, E and F), expression levels of CD98, GLUT1, HK2, or CPT1A did not differ significantly (Figure 8, G–J). Finally, the splenic STAT3^–/–^ CD4^+^ and CD8^+^ T cells did not differ from WT T cells in Mito-ROS production or Mito dysfunction (Figure 8, K and L). These results indicate that, although expression of GSH in splenic STAT3^–/–^ T cells is lower than in WT T cells, differences in GSH/Myc pathway activity and Myc protein expression are not statistically significant. In addition, STAT3 deficiency does not decrease Glyco-ATP or Mito-ATP production and does not increase Mito-ROS production or Mito-dysfunction in the splenic T cells. These results further indicate that lower PD-L1/PD-1 signaling in the splenic tissues allows the STAT3^–/–^ donor T cells to have functional metabolic reprogramming and relatively normal function.

### Mito-STAT3 deficiency alone does not effectively inhibit the GSH pathway or prevent acute GVHD.

**Figure 9:**
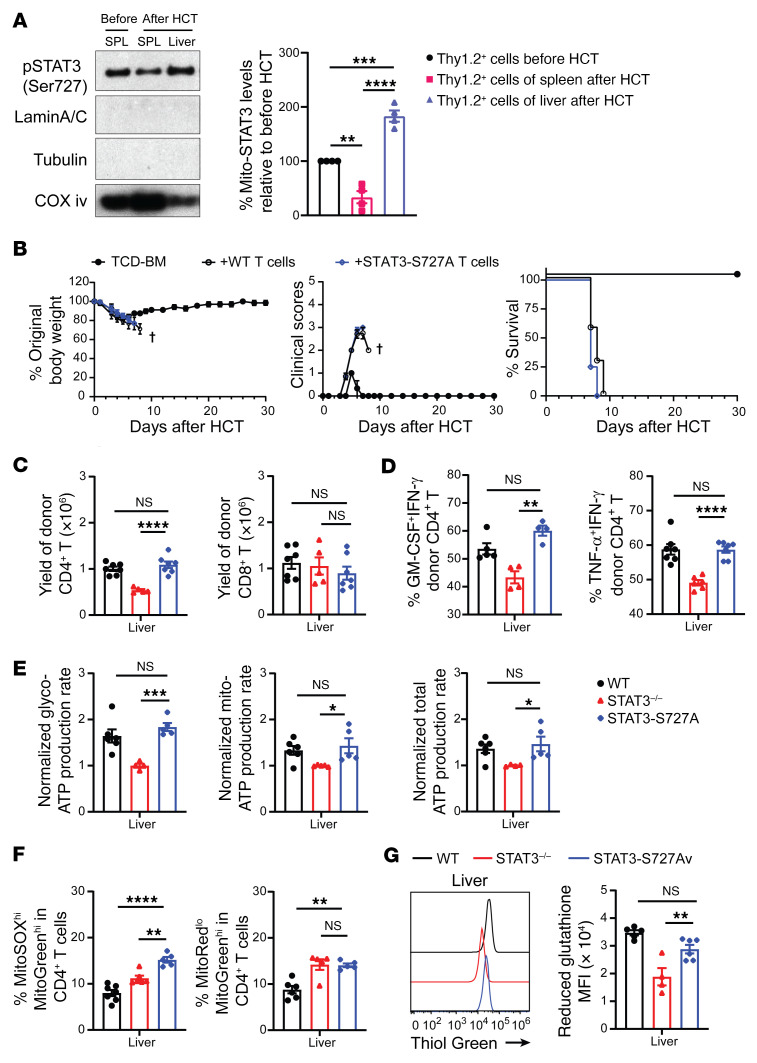
Mito-STAT3 deficiency alone does not reduce GSH synthesis or prevent acute GVHD. (**A**) Lethally irradiated BALB/c recipients were engrafted with TCD-BM (5 × 10^6^) and CD90.2^+^ T (1 × 10^6^) from WT C57BL/6 donors. Mitochondria of CD90.2^+^ T cells were isolated from donor spleen before HCT and from recipient spleen (SPL) and liver 6 days after HCT. Mitochondrial STAT3 levels were measured by immunoblotting. LaminA/C, tubulin, and COX iv were used as loading controls for nuclear, cytosol, and mitochondria, respectively. (**B**–**G**) Lethally irradiated BALB/c recipients were engrafted with TCD-BM (5 × 10^6^) from WT C57BL/6 donors and CD90.2^+^ T cells (1 × 10^6^) from WT or STAT3^–/–^ or STAT3-S727A C57BL/6 donors. On day 6 after HCT, lymphocytes from liver were isolated. (**B**) Plots of percentage original body weight, clinical GVHD score, and percentage survival. *n* = 3 (TCD-BM); *n* = 7 (TCD-BM+WT T); *n* = 8 (TCD-BM+STAT3-S727A T), combined from 2 replicated experiments. (**C**) Yields of donor CD4^+^ and CD8^+^ T cells. *n* = 5–7 combined from 2 replicates. (**D**) Percentages of TNF-α^+^IFN-γ^+^ and GM-CSF^+^IFN- γ^+^ Th1 cells. *n* = 4–7 combined from 2–3 replicates. (**E**) Experiments were performed as WT versus STAT3^–/–^ and STAT3^–/–^ versus STAT3-S727A. Relative Glyco-ATP, Mito-ATP, and total ATP production rates are shown, using the mean of STAT3^–/–^ T cells as the reference in each experiment. *n* = 4–6 combined from 2 replicates. (**F**) Percentages of MitoSOX^hi^MitoGreen^hi^ CD4^+^ T cells and MitoRed^lo^MitoGreen^hi^ CD4^+^ T cells from different groups are compared. (**G**) Representative flow cytometry pattern of reduced GSH and means ± SEM of MFI. *n* = 4–6 combined from 2 replicates. Data are represented as mean ± SEM. *P* values were calculated by 1-way ANOVA (**A** and **C**–**G**). NS, *P* ≥ 0.05; *P < 0.05; **P < 0.01; ***P < 0.001; ****P < 0.0001.

Compared with T cells from the donor spleen before HCT, T cells from the recipient spleen on day 6 after HCT had lower expression of pSTAT3-Ser727 in the mitochondria (Mito-STAT3), but those from the recipient liver had higher expression of Mito-STAT3 (Figure 9A). Therefore, we determined whether Mito-STAT3 deficiency alone is sufficient to prevent acute GVHD by testing T cells from donors with mutated Mito-STAT3 S727A. The STAT3 S727A mutation disrupts a variety of functions in mitochondria, but not elsewhere (56). Accordingly, donor T cells from Mito-STAT3–deficient STAT3-S727A mice or control WT littermates were engrafted together with TCD-BM cells from WT donors. As judged by percentage of body weight changes, clinical GVHD score, survival, and histopathology, the severity of acute GVHD did not differ between recipients given STAT3-S727A T or WT T cells (Figure 9B and Supplemental Figure 15). These results indicate that Mito-STAT3 deficiency alone in donor T cells does not prevent acute GVHD.

We explored mechanisms by comparing the expansion of WT, STAT3^–/–^ (pan-STAT3 deficient), and STAT3-S727A (Mito-STAT3 deficient) donor T cells infiltrating the liver and their cytokine expression and metabolic profiles on day 6 after HCT. Pan-STAT3 deficiency decreased the yield of CD4^+^ T cells in the liver, but did not affect the yield of CD8^+^ T cells, while Mito-STAT3 deficiency alone had no effect on the yield of either T cell subset in the liver (Figure 9C). Pan-STAT3 deficiency decreased the percentage of pathogenic TNF-α^+^IFN-γ^+^ and GM-CSF^+^IFN-γ^+^ Th1 cell subsets among CD4^+^ T cells and decreased their production of Mito-ATP and total ATP (Figure 9, D and E, and Supplemental Figure 16, A and B). Mito-STAT3 deficiency did not affect the percentage of pathogenic Th1 cells among CD4^+^ T cells or their production of Glyco-ATP, Mito-ATP, or total ATP. Although Pan-STAT3 deficiency and Mito-STAT3 deficiency both increased Mito-ROS production and Mito dysfunction (Figure 9F and Supplemental Figure 16C), GSH expression was significantly decreased by Pan-STAT3 deficiency, but not by Mito-STAT3 deficiency (Figure 9G).

Mito-STAT3 deficiency did not affect the percentage of GM-CSF^+^IFN-γ^+^CD8^+^ T cells, but decreased their expression of granzyme B expression, which was similar to the effect of pan-STAT3 deficiency on granzyme B expression in CD4^+^ T cells (Supplemental Figure 17, A and B). Mito-STAT3 deficiency increased Mito-ROS production and Mito dysfunction in CD8^+^ T cells (Supplemental Figure 17C). GSH expression was significantly decreased by Pan-STAT3 deficiency, but not by Mito-STAT3 deficiency (Supplemental Figure 17D). These results indicate that Mito-STAT3 deficiency in donor CD4^+^ and CD8^+^ T cells increases Mito-ROS production and Mito dysfunction, but does not cause significant inhibition of GSH/Myc pathways or dysfunction of metabolic reprogramming of alloactivated T cells, such that these donor T cells have no impairment of their ability to induce acute GVHD.

### Degradation of STAT3 in donor T cells prevents acute GVHD in a PD-1–dependent manner.

**Figure 10:**
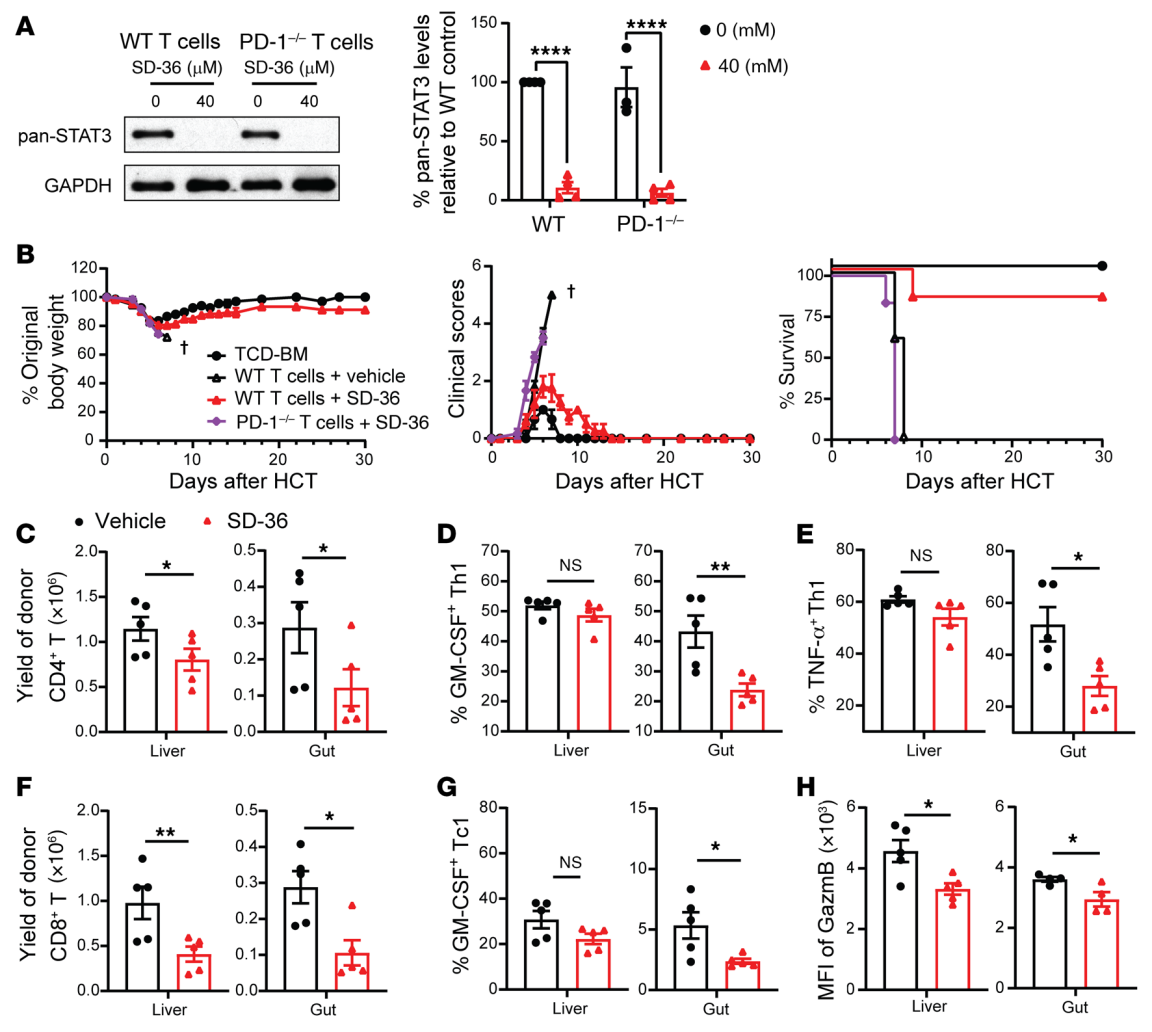
Degradation of STAT3 in donor T cells prevents acute GVHD in a PD-1–dependent manner. (**A**) Splenic T cells from WT or PD-1^–/–^ C57BL/6 mice were treated with or without 40 μM SD-36. T cell expression of STAT3 was measured by immunoblotting after 24 hours. Representative immunoblotting patterns and means ± SEM of expression levels are shown. (**B**) Irradiated BALB/c recipients were engrafted with TCD-BM (5 × 10^6^) from WT C57BL/6 donors alone or with purified T cells (1 × 10^6^) from spleens of WT or PD-1^–/–^ C57BL/6 donors after culture in medium containing 40 μM SD-36 for 24 hours in vitro. Recipients were treated with SD-36 (50 mg/kg, i.v.) or vehicle on days 0 and 3 after HCT. Plots of percentages of original body weight and clinical GVHD score and percentage survival are shown. *n* = 3 (TCD-BM); *n* = 5 (WT T cells+vehicle); *n* = 6 (WT T cells+SD-36); *n* = 6 (PD-1^–/–^ T cells+SD36) combined from 2 replicate experiments. (**C**–**H**) On day 6 after HCT, lymphocytes from the liver and gut were analyzed with flow cytometry. *n* = 4–5 combined from 2 replicate experiments. (**C**) Yield of CD4^+^ donor T cells are shown. (**D** and **E**) Percentage of GM-CSF^+^IFN-γ^+^ and TNF-α^+^IFN-γ^+^ among CD4^+^ T cells. (**F**) Yield of CD8^+^ donor T cells. (**G**) Percentages of GM-CSF^+^IFN-γ^+^ among CD8^+^ T cells are shown. *n* = 5 combined from 2 replicate experiments. (**H**) MFI of GzmB of CD8^+^ T cells is shown. *n* = 4–5 representing means ± SEM. *P* values were calculated by 2-way ANOVA (**A**) or unpaired 2-tailed Student *t* tests (**C**–**H**). NS, *P* ≥ 0.05; *P < 0.05; **P < 0.01; ****P < 0.0001.

STAT3-degrader SD-36 can effectively degrade STAT3 in tumor cells (57). Similarly, 24-hour culture in medium containing 40 μM SD-36 degraded STAT3 in WT and PD-1^–/–^ T cells (Figure 10A). To evaluate the effects of STAT3 degradation on GVHD, SD-36 or solvent-treated C57BL/6 WT or PD-1^–/–^ donor T cells (1 × 10^6^) were cotransplanted with TCD-BM cells (5 × 10^6^) into irradiated BALB/c recipients. Recipients were treated with SD-36 (50 mg/kg) or solvent via i.v. administration on days 0 and 3 after HCT. Solvent-treated WT T cells induced lethal acute GVHD, and all (5/5) recipients died within 10 days, while SD-36–treated WT T cells induced mild acute GVHD and most (5/6) survived for more than 30 days. In contrast, SD-36–treated PD-1^–/–^ T cells induced lethal acute GVHD, and all (6/6) died within 10 days (Figure 10B). Prevention of acute GVHD by SD-36 treatment of WT T cells was associated with lower serum concentrations of IFN-γ, TNF-α, and IL-6, but not IL-2 (Supplemental Figure 18A), and was confirmed by histopathology (Supplemental Figure 18, B and C). Treatment with SD-36 decreased the yield of CD4^+^ T cells in liver and gut and decreased the percentage of pathogenic GM-CSF^+^IFN-γ^+^CD4^+^ Th1 and TNF-α^+^IFN-γ^+^CD4^+^ Th1 cells in the gut but not in liver on day 6 after HCT (Figure 10, C–E). SD-36 treatment also decreased the yield of donor CD8^+^ T cells in the liver and gut, decreased the percentage of pathogenic GM-CSF^+^IFN-γ^+^Tc1 cells in the gut, but not in the liver, and decreased granzyme B expression in the liver and gut (Figure 10, F–H). These results indicate that, consistent with genetic deficiency of STAT3 in donor T cells, degradation of STAT3 in donor T cells by SD-36 also prevents acute GVHD in a donor T cell PD-1–dependent manner.

## Discussion

In the current studies, we demonstrate with murine models of GVHD that Pan-STAT3 deficiency (STAT3^–/–^), but not Mito-STAT3 deficiency, in donor T cells induces only mild and reversible acute GVHD while preserving GVL activity that eliminates BCL1 and even non-GVL susceptible ALL cells in a donor T cell dose-dependent manner. GVHD prevention requires GVHD target tissue expression of PD-L1 and T cell expression of PD-1. Lack of host tissue cells that express PD-L1 in lymphohematopoietic tissues preserves the GVL activity of STAT3^–/–^ donor T cells. The preserved expansion of STAT3^–/–^ T cells in the spleen is associated with continued production of Glyco-ATP and Mito-ATP without significant inhibition of GSH/Myc pathways. The absence of recipient cells that express PD-L1 in lymphohematopoietic tissues early after HCT (15) may help to preserve the GVL activity of STAT3^–/–^ donor T cells. In contrast, continued expression of PD-L1 in GVHD target parenchymal tissues triggers PD-1 signaling in the STAT3^–/–^ T cells to downregulate GSH/Myc pathways, leading to impaired metabolic reprogramming, T cell dysfunction, and GVHD prevention. In addition, degradation of Pan-STAT3 by SD-36 (57) in donor T cells early after HCT prevents acute GVHD in a T cell PD-1–dependent manner. Our studies provide insights into mechanisms whereby STAT3 deficiency regulates the outcome of tissue PD-L1/PD-1 signaling in alloreactive T cells. These results provide a scientific basis for targeting STAT3 in donor T cells as an approach to preventing GVHD while preserving GVL activity.

Although PD-1 signaling increased Mito-ROS production and Mito dysfunction in WT T cells (49), PD-L1/PD-1 signaling in GVHD target tissue infiltrating WT T cells did not sufficiently inhibit the GSH/Myc pathway to cause dysfunctional metabolic reprogramming and T cell dysfunction to a degree that prevents GVHD. PD-1 signaling in WT T cells reduces glycolysis, but upregulates CPT1A expression and promotes lipolysis and FAO to sustain T cell function (49). In contrast, we observed that PD-1 signaling in STAT3^–/–^ T cells reduced both Glyco-ATP (glycolysis) and Mito-ATP (FAO) and also reduced GSH synthesis, with reduced expression of CD36 and CD98 to a degree that induced metabolic dysfunction and deletion of T cells infiltrating the liver and gut, thereby preventing GVHD. Therefore, simultaneous tissue PD-L1/PD-1 signaling and STAT3 deficiency in donor T cells are required for effective prevention of GVHD.

With Mito-STAT3 deficiency alone, PD-1 signaling did not inhibit the GSH/Myc pathway or prevent acute GVHD. Tumor cells with dysfunctional STAT3-S727A mutation in mitochondria have decreased GSH expression, increased Mito-ROS production, and Mito dysfunction that increased tumor cell apoptosis (25, 58). In contrast, we observed that Mito-STAT3 S727A T cells from GVHD target tissues did not significantly reduce GSH expression, although STAT3^–/–^ T cells and Mito-STAT3 S727A T cells did not differ in Mito-ROS production and Mito dysfunction. The GVHD target tissue–infiltrating STAT3-S727A T cells had preferential expansion of CD4^+^ T cells, but did not differ from WT T cells in their production of Glyco-ATP, Mito-ATP, and total ATP, leading to severe GVHD. The differences between Mito-STAT3 deficiency and pan-STAT3 deficiency highlight the reductions in CD98 and GCLC expression and GSH synthesis as essential mechanisms whereby STAT3 deficiency prevents GVHD. These results are consistent with reports that effective inhibition of the GSH/Myc pathway is important for prevention of tissue inflammation (35). These results also suggest that, unlike in certain tumor cells, Mito-STAT3 deficiency in donor T cells that infiltrate GVHD target tissues cannot augment their PD-1–dependent apoptosis to a degree that prevents acute GVHD.

Regulation of ROS production and metabolic reprogramming by PD-1 signaling and STAT3 deficiency in donor T cells is most likely through GSH. ROS is a side product of oxidative phosphorylation in mitochondria (59) and has complex effects during GVHD pathogenesis (20, 60). Trx1 and GSH both play important roles in buffering ROS and regulating alloreactive T cell expansion and survival. Donor T cells with Trx1 transgene expression had reduced ROS production, less severe acute GVHD, and preserved GVL effects (60). Pan-STAT3 deficiency in donor T cells augments Mito-ROS production and reduces GSH synthesis, but had no detectable effect on Trx1 expression. By scavenging ROS in WT T cells, GSH prevents oxidative damage to enzymes in the Myc pathway that enable T cell metabolic reprogramming, as reported by others (35). In contrast, inadequate scavenging of increased Mito-ROS production by decreased GSH synthesis in STAT3-deficient T cells allows oxidative damage to enzymes in the Myc pathway, thereby preventing metabolic reprogramming. Although Trx1 and GSH both scavenge ROS, they may be expressed in different types of cells (i.e., lymphocytes versus innate immune cells) or have different timing of expression (i.e., before T cell activation versus after T cell activation) during GVHD pathogenesis. This issue needs to be addressed in future studies.

Increased ROS production does not fully explain the ability of donor T cell STAT3 deficiency to prevent GVHD. Although antioxidant NAC was able to improve STAT3^–/–^ T cell function in an in vitro MLR assay in a PD-L1/PD-1 signaling–dependent manner, in vivo administration of antioxidant NAC or MnTBAP did not augment GVHD induced by STAT3^–/–^ T cells, although previous studies have shown that antioxidants augment acute GVHD induced by WT T cells (20). This difference between WT and STAT3^–/–^ T cells suggests that STAT3 deficiency in T cells augments inhibition of GSH/Myc pathways that play a critical role in metabolic reprogramming by downregulating expression of Myc, CD36, and CD98 in the T cells, as mentioned above.

Differential metabolic profile of CD4^+^ and CD8^+^ T cell subsets in the spleen of recipients might contribute to maintenance of GVL activity mediated by STAT3^–/–^ T cells. In the spleen, STAT3^–/–^CD4^+^ T cells downregulate GSH metabolism, glycolysis/gluconeogenesis, and oxidative phosphorylation, although the reduction was less than that of STAT3^–/–^CD4^+^ T cells in the liver, reflecting lower PD-L1/PD-1 signaling that exists in the spleen. In contrast, STAT3^–/–^CD8^+^ T cells in the spleen downregulate only oxidative phosphorylation. These results suggest that persistent upregulation of GSH metabolism, glycolysis/gluconeogenesis, and fatty acid metabolism in STAT3^–/–^CD8^+^ T cells in lymphohematopoietic tissues helps to preserve their GVL activity. These differential metabolic profiles in STAT3^–/–^ CD4^+^ and CD8^+^ T cells in the spleen may result from their differential expression of PD-1, CD80, and PD-L1 and need to be addressed in future studies. We reported that CD8^+^ T cells in the lymphoid tissues expressed higher levels of PD-L1 and CD80, but lower levels of PD-1, and PD-L1/CD80 interaction augmented donor CD8^+^ T expansion in the lymphoid tissues early after HCT (10).

Besides anatomic tissue microenvironment (i.e., lymphoid tissue versus parenchymal tissue), the microenvironment of tissue inflammation may also regulate the effects of STAT3 deficiency on the regulation of T cell metabolic programing. STAT3-deficient CD8^+^ T cells have enhanced antitumor function with increased production of IFN-γ and granzyme in the chronic inflammatory environment of tumor tissues (61–63), which was associated with upregulated expression of Glut-1 and enhanced glycolysis and downregulated expression of CPT1B and reduced FAO (63). In contrast, we showed that STAT3 deficiency in donor CD8^+^ T cells in GVHD target tissues of acute inflammation resulted in reduced glycolysis and FAO. Our observation is consistent with a previous report indicating that allogeneic T cells in the acute GVHD target tissues, but not syngeneic T cells, showed a ROS^hi^PD-1^hi^ phenotype (20). Although our data indicate that targeting STAT3 in donor T cells can prevent acute GVHD while preserving GVL effect, whether targeting STAT3 in donor T cells could control established acute or chronic GVHD without inhibiting GVL activity is not yet known and should be addressed in future studies.

Other investigators have done studies to prevent acute GVHD by targeting glycolysis (30, 51) and OXPHOS/glutaminolysis (31) in donor T cells. Those reports all used splenic T cell metabolic profiles to reflect the metabolic reprogramming of donor T cells in allo-HCT recipients (30, 31, 51, 64). Consistent in part with these studies, we observed a simultaneous burst of glycolysis and OXPHOS in donor T cells early after allogeneic HCT; however, the kinetics of glycolysis and OXPHOS differed, and OXPHOS appeared to be dominant as time went on. More importantly, we observed that PD-L1/PD-1 signaling mainly affected the metabolic profile of donor T cells in GVHD target tissues but not in the spleen, and a synergism between tissue-specific PD-L1/PD-1 signaling and STAT3 deficiency in donor T cells contributed to prevention of GVHD.

Our studies with STAT3^–/–^ T cells have demonstrated that specific targeting of STAT3 in donor T cells can preserve strong GVL activity while preventing GVHD. The observation that degradation of STAT3 in donor T cells by in vitro culture with STAT3 degrader SD-36 prevented acute GVHD has demonstrated the translational potential of approaches that specifically target STAT3 in donor T cells to prevent GVHD while preserving GVL activity. Although STAT3 degradation by SD-36 can induce tumor cell apoptosis (57), STAT3 also has multiple vital biological functions in a variety of tissues and cell types. For example, STAT3 signaling supports the regeneration of epithelial cells in the gut and skin (65, 66). Therefore, future studies will be needed to determine whether SD-36 and other approaches that target STAT3 (67–69) can preserve GVL activity while preventing GVHD as well as to determine the severity of side effects caused by in vivo administration of SD-36.

In summary, in the GVHD target tissues, STAT3 is present in the nucleus and mitochondria of activated WT donor T cells. Tissue PD-L1 interaction with PD-1 on T cells triggers low-level production of Mito-ROS and allows GSH/Myc pathways to promote metabolic reprogramming with increased Glyco-ATP and Mito-ATP, leading to robust T cell expansion and survival and induction of GVHD. In contrast, the absence of STAT3 in the nucleus and mitochondria allows PD-1 signaling to trigger high-level production of Mito-ROS and to inhibit the GSH/Myc pathway and metabolic reprogramming. The lower production of both Glyco-ATP and Mito-ATP causes T cell anergy, exhaustion, and apoptosis, thereby preventing GVHD. In recipient lymphohematopoietic tissues, due to the lack of PD-L1 triggering PD-1 signaling, the absence of STAT3 in the nucleus and mitochondria limits Mito-ROS production and still allows GSH/Myc pathways to promote metabolic reprogramming with increased Glyco-ATP and Mito-ATP production, leading to robust T cell expansion and survival to mediate GVL activity. Our results suggest that targeting STAT3 in donor T cells before or early after HCT could represent an effective approach for preventing GVHD while preserving GVL effects.

## Methods

### Mice.

BALB/c and C57BL/6 mice were purchased from the National Cancer Institute laboratories. PD1^–/–^ C57BL/6 breeders were provided by Haidong Dong (Mayo Clinic, Rochester, Minnesota, USA) with approval of Dr. Tasuku Honjo (Tokyo University, Tokyo, Japan). C57BL/6 STAT3^fl/fl^ and CD4^Cre^ breeder mice were provided by Hua Yu (City of Hope, Duarte, California, USA). STAT3^–/–^PD-1^–/–^ mice were generated by crossing STAT3^–/–^ with PD-1^–/–^ mice. All mice were kept in a specific pathogen–free room in City of Hope-Animal Resources Center. Splenic cells from STAT3-S727A and control mice were provided by M. Isbell (Department of Pathology, NYU Grossman School of Medicine).

### Experimental procedures.

Experimental procedures including (a) induction and assessment of GVHD, (b) isolation of lymphocytes from GVHD target tissues, (c) flow cytometry analysis and cell sorting, (d) and histopathology, histoimmunochemistry, and histoimmunofluorescent staining were as described in previous publications (10, 14, 15) and Supplemental Methods.

### Statistics.

Data are represented as mean ± SEM. Comparison of percentage survival in groups was analyzed by log-rank test. Two-group means comparison was analyzed by using an unpaired, 2-tailed Student’s *t* test. For evaluation of 3 means, we used 1-way ANOVA for multiple comparisons. For evaluation of 2 independent variables on a dependent variable, we used 2-way ANOVA for multiple comparisons (Prism, version 7). Adjusted *P* values in GSEA plots of MYC target V2 pathway were calculated by using the clusterProfile, version 3.16.1, and msigdbr, version 7.4.1, packages in R. Two-sided *P* values of less than 0.05 and adjusted GSEA *P* values of less than 0.25 were considered statistically significant.

### Study approval.

All animal procedures were approved by the IACUC of the Beckman Research Institute of City of Hope.

### Data availability.

The RNA sequencing data have been deposited in the GEO database (GSE215166).

## Author contributions

QL designed and performed experiments, acquired and analyzed data, and prepared the manuscript. X Wang designed and performed experiments and acquired and analyzed data in part. QS assisted with experimental design, data analysis, and manuscript preparation. X Wu and HQ performed RNA-Seq and data analysis. DY provided advice on statistical analysis. SY, UN, XK, KC, JT, MZ, EL, and BW assisted with experiments. PJM provided advice on experimental design and critical review and edited the manuscript. XZ served as QL and X Wang’s PhD mentor and helped with advising on QL and X Wang’s research activities. IJM and DL provided the STAT3-S727A mutated mice. DZ designed and supervised the research and wrote the manuscript.

## Supplementary Material

Supplemental data

## Version 1 08/01/2023

Electronic publication
