# Supplementary material for: Donor T cell STAT3 deficiency enables tissue PD-L1–dependent prevention of graft-versus-host disease while preserving graft-versus-leukemia activity
Source: J Clin Invest. 2023 Aug 1;133(15):e165723. doi: 10.1172/JCI165723 (PMC10378157; doi:10.1172/JCI165723)
Supplement: Supplemental data [file jci-133-165723-s110.pdf]

Supplementary Materials for

Donor T cell STAT3 deficiency enables tissue PD-L1-dependent prevention of graft-versus-host-disease while preserving graft-versus-leukemia activity.

Qinjian Li[1,2,3♣], Xiaoqi Wang[1, 2, 3♣], Qingxiao Song[1, 2, 4♣], Shijie Yang[1, 2, 3♣], Xiwei Wu[5], Dongyun Yang[2], Isabelle Marié[6], Hanjun Qin[5], Moqian Zheng[1, 2], Ubaydah Nasri[1, 2], Xiaohui Kong[1,2], Bixin Wang[1, 2, 4], Elizabeth Lizhar[1], Kaniel Cassady[1, 2], Josh Tompkins[1], David Levy[6], Paul J. Martin[7], Xi Zhang[3], Defu Zeng[1,2]

Corresponding authors: Defu Zeng, MD, Beckman Research Institute of City of Hope, 1500 East Duarte Road, Duarte, CA 91010. Phone: 626-218-3587. E-mail: dzeng@coh.org. or Xi Zhang, MD, PhD, Medical Center of Hematology, State key Laboratory of Trauma, Burn and Combined Injury. Army Medical University, Chongqing, China. E-mail: zhangxxi@sina.com

Files include Supplemental Figures and Tables as well as supplemental materials and methods.

I. **Supplemental Figures**

II. **Supplemental Materials and Methods**

I. Supplemental Figures

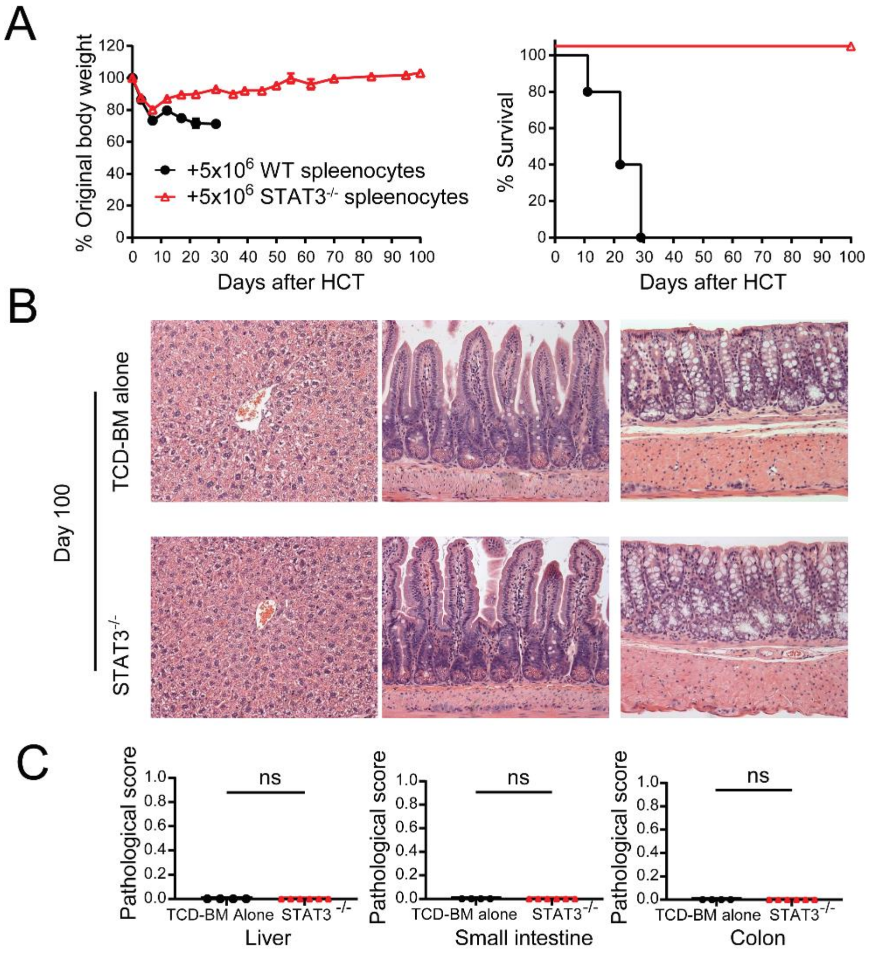

Figure S1 (Supplemental to Figure 1). STAT3 deficiency in donor T cells prevents both acute and chronic GVHD. Lethally irradiated BALB/c recipients were engrafted with TCD-BM (5 x 10^6) from WT C57BL/6 donors and splenocytes (5 x 10^6) from STAT3$^{-/-}$ or WT C57BL/6 donors. **(A)** Plots of %bodyweight changes and %survival are shown. n = 10 combined from two replicated experiments. **(B)** Histopathology of liver (left), small intestine (middle) and colon (right) evaluated on day 100 after HCT. Representative micrographic photos of liver, small intestine, and colon (original magnification, ×200) are shown. **(C)** Means ± SEM of pathological score for liver, small intestine and colon are shown. n = 4-6 per group combined from two replicate experiments. Data represent means ± SEM. p values were calculated using unpaired two-tailed Student's t test for mean comparisons. $^{ns}p \geq 0.05$.

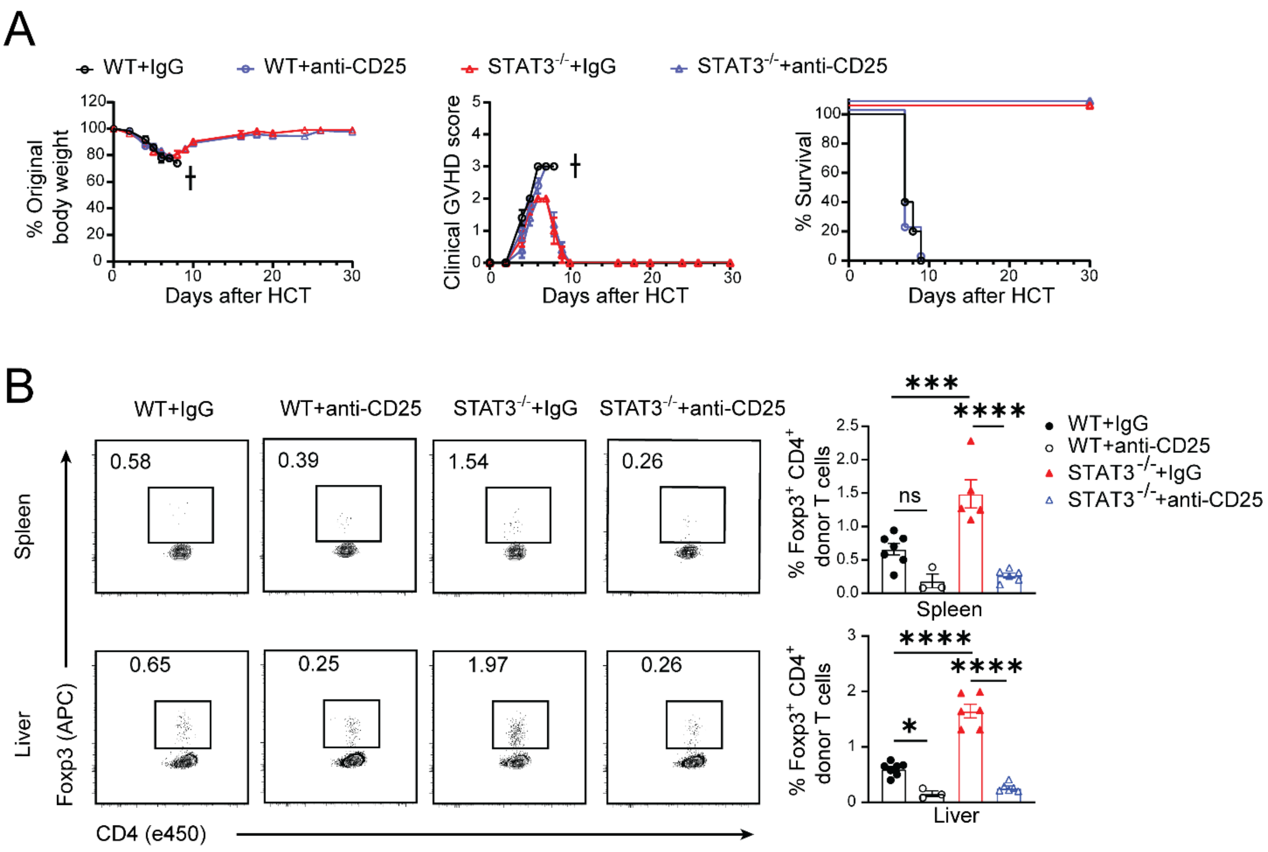

Figure S2. Prevention of GVHD by STAT3-deficiency in donor T cells does not depend on Treg cells. Lethally irradiated BALB/c recipients were engrafted with TCD-BM cells (5 x 10^6) from WT C57BL/6 donors and CD90.2$^+$ T cells (1 x 10^6) from WT or STAT3$^{-/-}$ C57BL/6 donors. Recipients were treated with anti-CD25 (PC-61.5.3) or control IgG (200 μg/mouse, i.p) on days 0, 2, 4, 6. **(A)** Plots of %body weight and clinical GVHD score as well as %survival are shown. *n* = 5 (WT + IgG), *n* = 5 (WT + anti-CD25), *n* = 4 (STAT3$^{-/-}$ + IgG), *n* = 5 (STAT3$^{-/-}$ + anti-CD25) combined from two replicated experiments. **(B)** On day 6 after HCT, lymphocytes from spleen, liver were isolated. Representative flow cytometry pattern and Means ± SEM of %Foxp3$^+$ CD4$^+$ T cells from spleen and liver are shown, *n* = 3-7 combined from two replicated experiments. "†" indicates death. *p* values were calculated by one-way analysis of variance (B). $^{ns}p \geq 0.05$, $*p < 0.05$, $***p < 0.001$, $****p < 0.0001$.

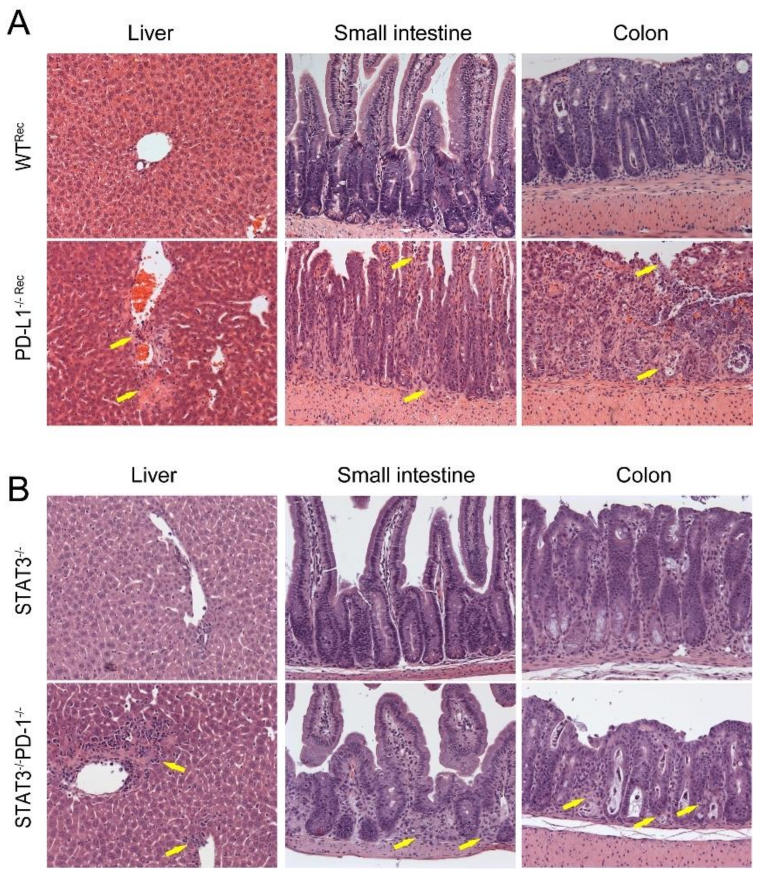

Figure S3 (Supplemental to Figure 4). Representative micrographic photos of liver, small intestine, and colon for Figure 4D **(A)** and Figure 4F **(B)** (arrows point to infiltrating T cells or tissue damage area, original magnification, ×200).

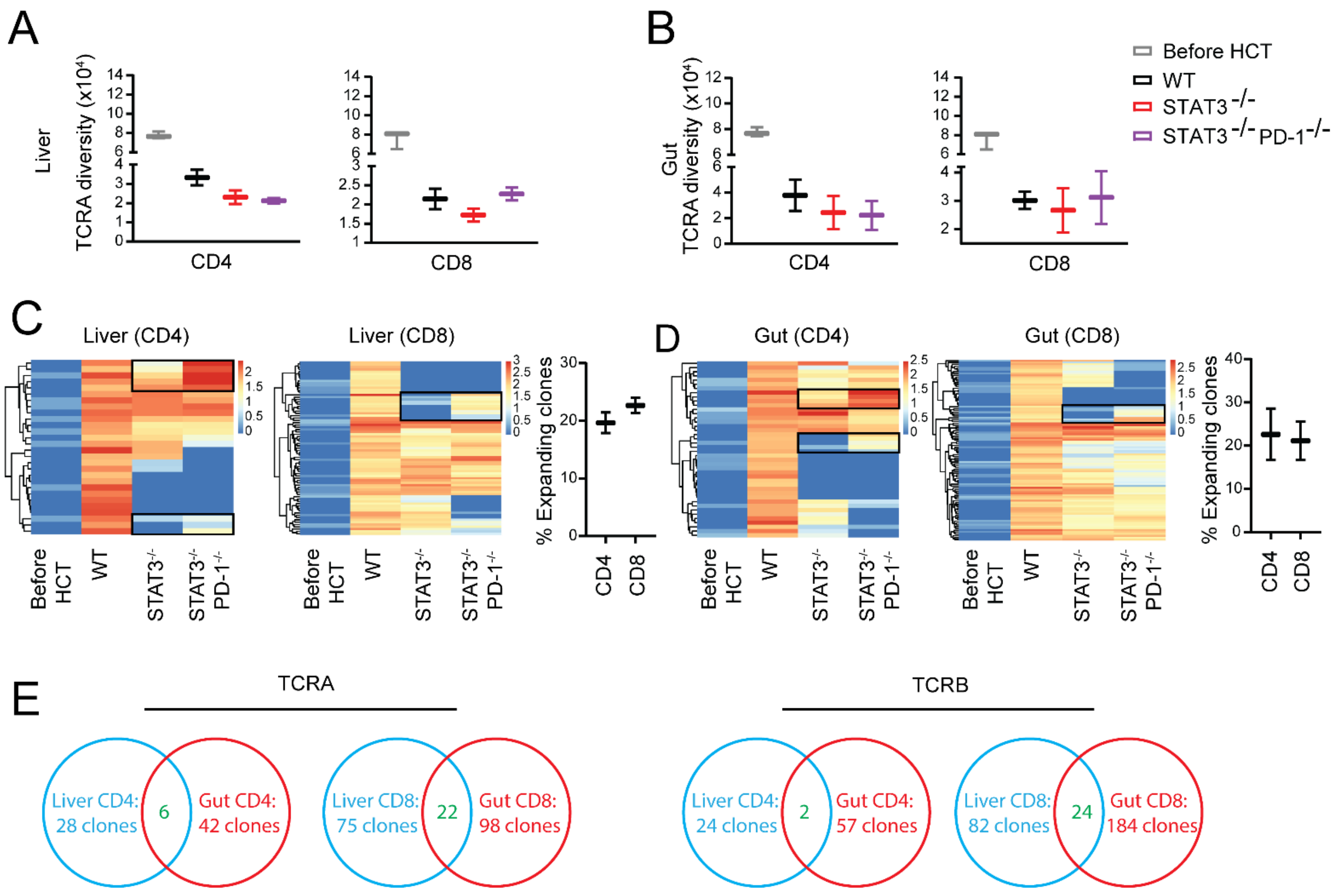

Figure S4 (Supplemental to Figure 5). TCR-repertoire and clonotype changes determined by TCRA-Seq analysis. The same donor T cells from Figure 5 were analyzed for donor T cell diversity and clonotype using TCRA-Seq. (**A-B**) CDR3 diversity of TCRA of CD4 and CD8 in **(A)** liver and **(B)** gut were compared. (**C-D**) Heat maps of TCRA in **(C)** liver and **(D)** gut were compared. The numbers are log (base 10 with offset of 1) transformed TCR frequency, which have been normalized to counts per million. Each sample contained lymphocytes from 3 recipients. **(E)** Venn diagram showing expanded TCRA and TCRB clonotypes in the liver and gut.

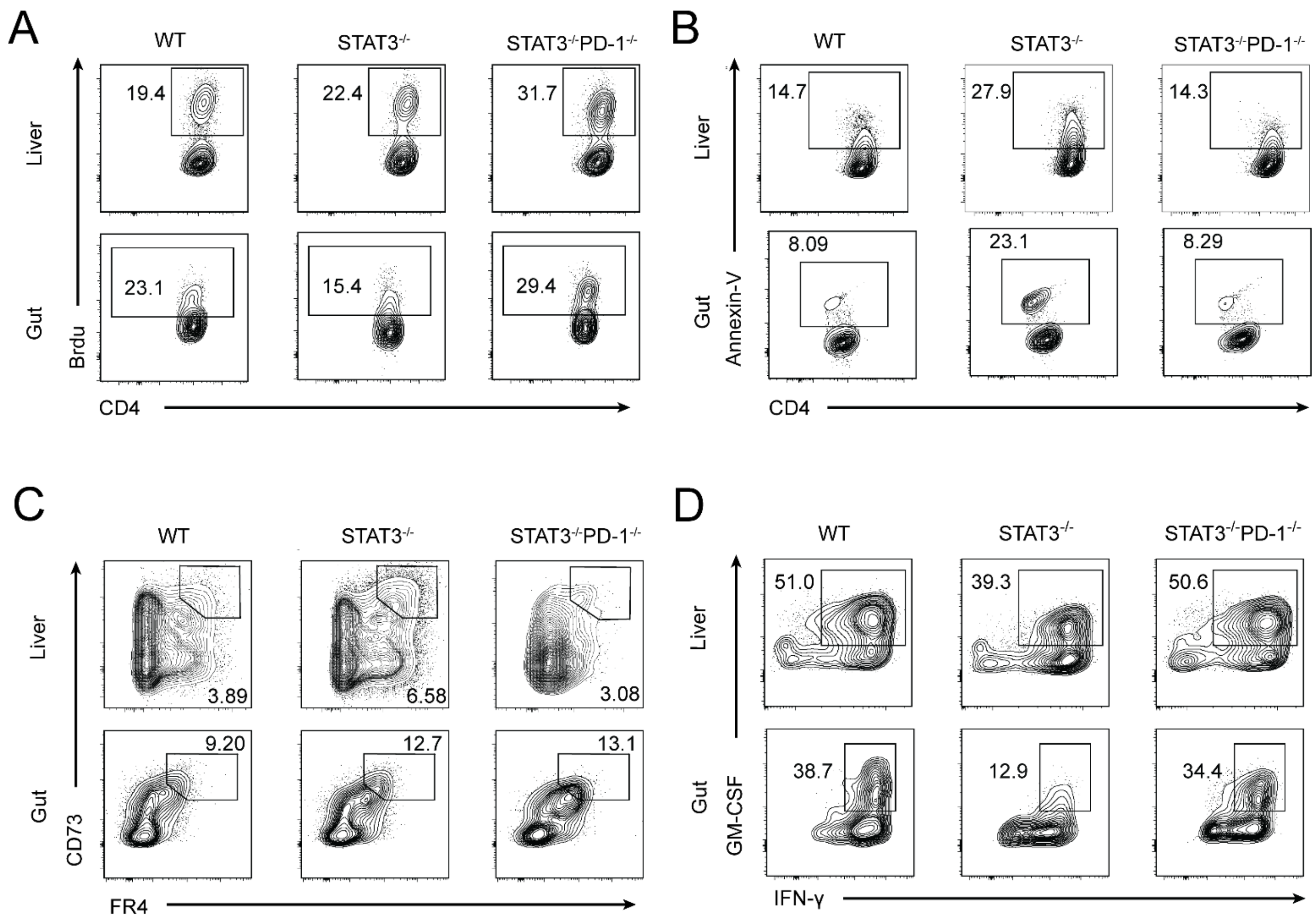

Figure S5 (Supplemental to Figure 5). (A-D) Representative flow cytometry patterns for Figure 5 (E-H).

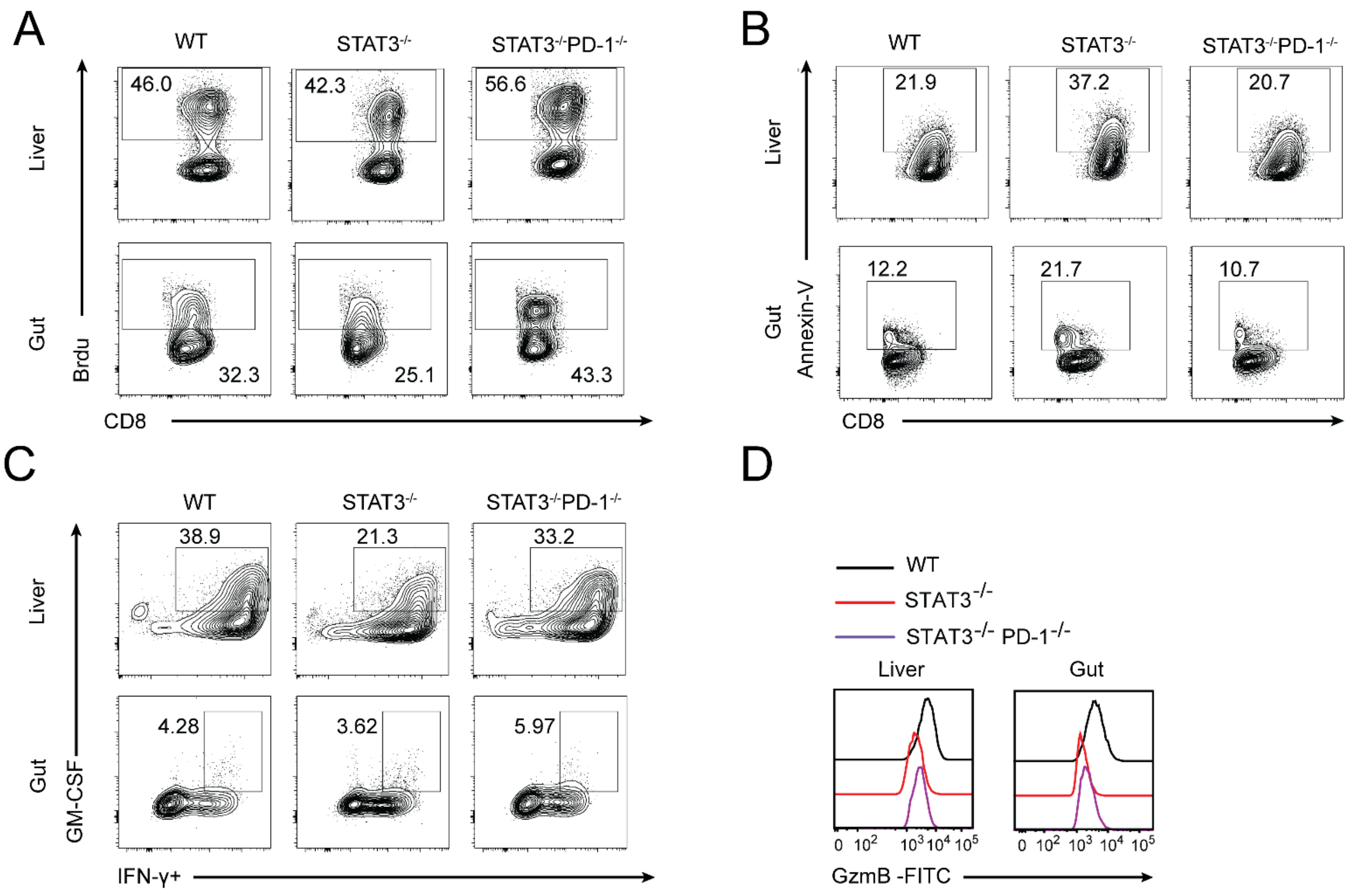

Figure S6 (Supplemental to Figure 5). Representative flow cytometry patterns for Figure 5 (I-L).

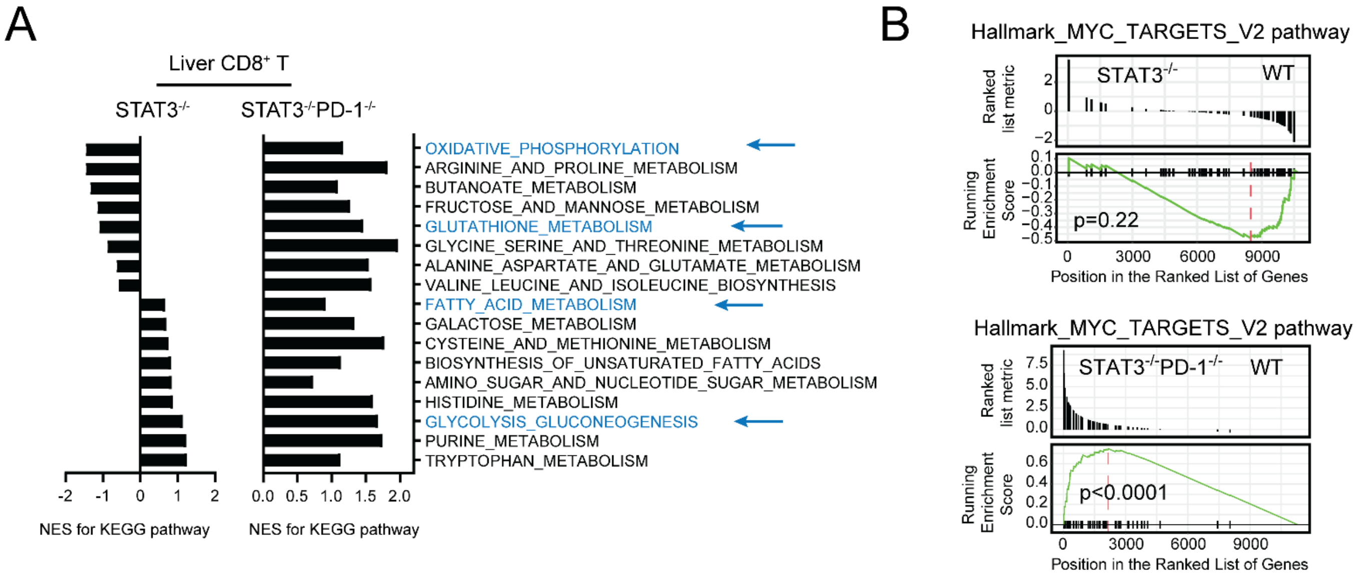

Figure S7 (Supplemental to Figure 6). Stat3 deficiency in donor T cells augments PD-1-mediated inhibition of GSH-Myc pathways. CD8$^+$ T cells from the same liver tissue of recipients used in Fig. 6 were analyzed with RNA-seq. **(A)** NES of KEGG Pathway activity of CD8$^+$ T cells, setting WT CD8$^+$ T cells as the reference. **(B)** GSEA plots of MYC target V2 pathway related gene set expression in STAT3$^{-/-}$ and STAT3$^{-/-}$PD-1$^{-/-}$ CD8$^+$ T cells compared to WT T cells.

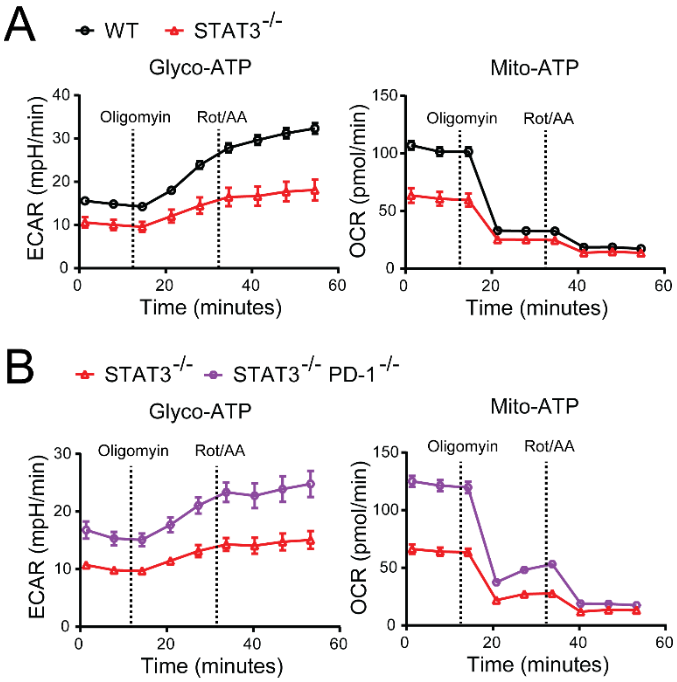

Figure S8. Representative patterns for Seahorse XF Real-Time ATP Rate Assay. On day 6 after HCT, $CD90.2^+$ donor T cells from liver were isolated for the Seahorse Assay. Oligomycin, or antimycin/rotenone (Rot/AA) treatments were given at the indicated time points. **(A-B)** Representative OCR (Mito-ATP) and ECAR (Glyco-ATP) curves for Seahorse XF Real-Time ATP Rate Assay. Measurement details are described in Supplementary Methods.

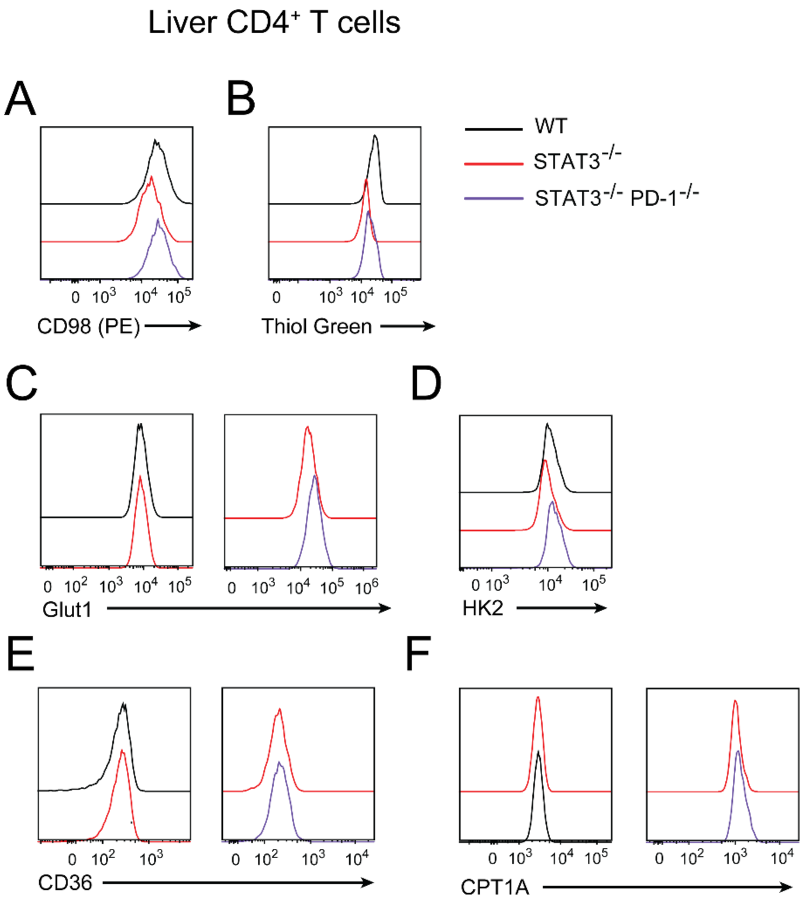

Figure S9 (Supplemental to Figure 6). (A-F) Representative flow cytometry patterns for Fig. 6 (F-G, I-J, L-M).

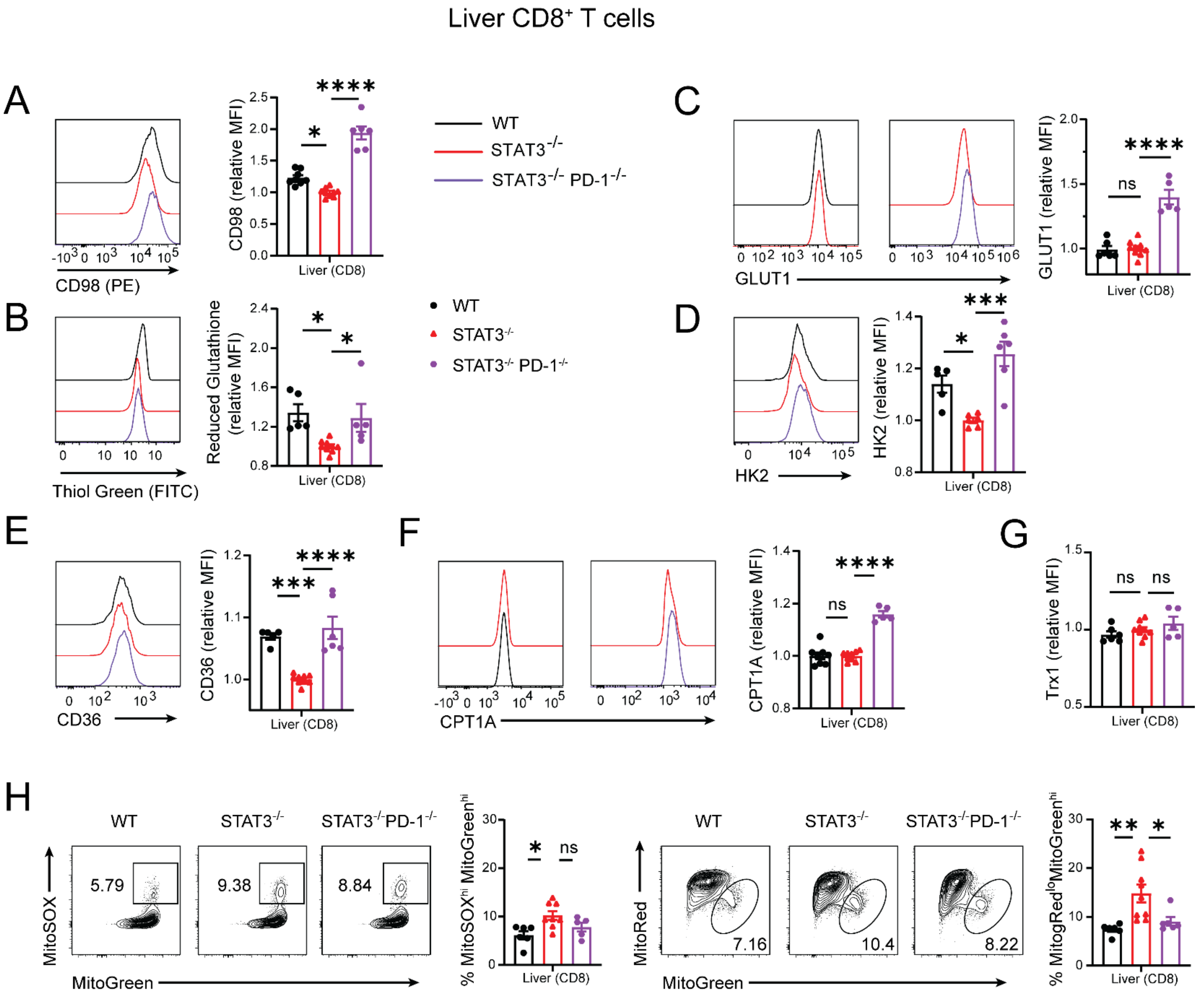

Figure S10 (Supplemental to Figure 6). Analysis of the CD8⁺ T cells from the same liver tissue of recipients used in Fig. 6 **(A-B)** MFI of CD98 and reduced GSH in CD8⁺ T cells; **(C-D)** MFI of GLUT1 and HK2 in CD8⁺ T cells; **(E-F)** MFI of CD36 and CPT1A **(G)** MFI of Trx1. **(H)** Representative flow cytometry pattern and means ± SEM of %MitoSOX^{hi}MitoGreen^{hi} and MitoRed^{lo}MitoGreen^{hi}. Separate experiments of WT versus STAT3$^{-/-}$ and STAT3$^{-/-}$ versus STAT3$^{-/-}$PD-1$^{-/-}$ were performed. n = 5-9 combined from at least 2 replicate experiments. Data represent means ± SEM. p values were calculated by one way ANOVA. $^{ns}p \geq 0.05$, $^{*}p < 0.05$, $^{**}p < 0.01$, $^{***}p < 0.001$, $^{****}P < 0.0001$.

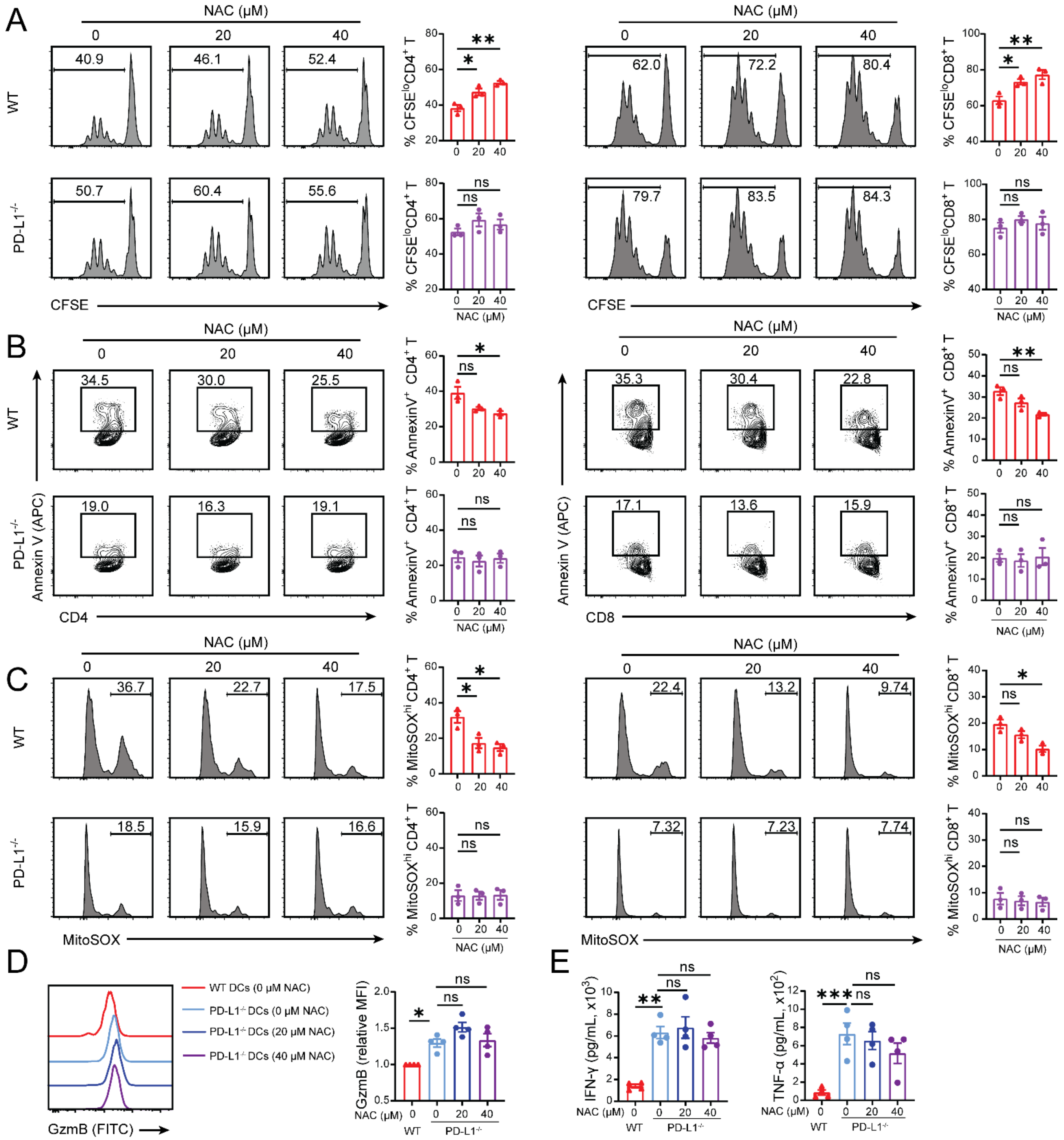

Figure S11 (Supplemental to Figure 7). Impact of antioxidant NAC on donor Stat3$^{-/-}$ T cell function is host PD-L1-dependant. CSFE-labeled STAT3$^{-/-}$ CD90.2^{+} T cells from donor C57BL/6 were co-cultured with irradiated WT or PD-L1$^{-/-}$ host BALB/c DCs in mixed lymphocyte reactions. The cells were treated with NAC at 0, 20, or 40μM on day 0. **(A)** T cells were collected on day 4 after co-culture for flow cytometric analysis of CD8^{+} and CD4^{+} T cells, respectively. Representative flow cytometry pattern and Means ± SEM of %CFSE^{lo}H-2Kb^{+}TCR-β^{+}CD4^{+} and CD8^{+} cells are shown. *n* = 3 per group combined from 3 replicated experiments. **[B-D]** The cells or supernatant were collected on day 4 for analysis. (B) Representative flow cytometry pattern and Means ± SEM of %AnnexinV^{+}CD4^{+} and CD8^{+} T cells are shown. *n* = 3 per group combined from 3 replicated experiments. (C) Representative flow cytometry pattern and Means ± SEM of %MitoSOX^{hi}CD4^{+} and CD8^{+} T cells are shown. *n* = 3 per group combined from 3 replicated experiments. (D) Representative flow cytometry pattern and Means ± SEM of MFI of GzmB are shown. *n* = 4 per group combined from 4 replicated experiments. Data represent Means ± SEM. (E) Concentrations of INF-γ and TNF-α in supernatants. *n* = 4 per group combined from 4 replicated experiments. *p* values were calculated using one-way analysis of variance. $^{ns}p \geq 0.05$, $*p < 0.05$, $**p < 0.01$, $***p < 0.001$.

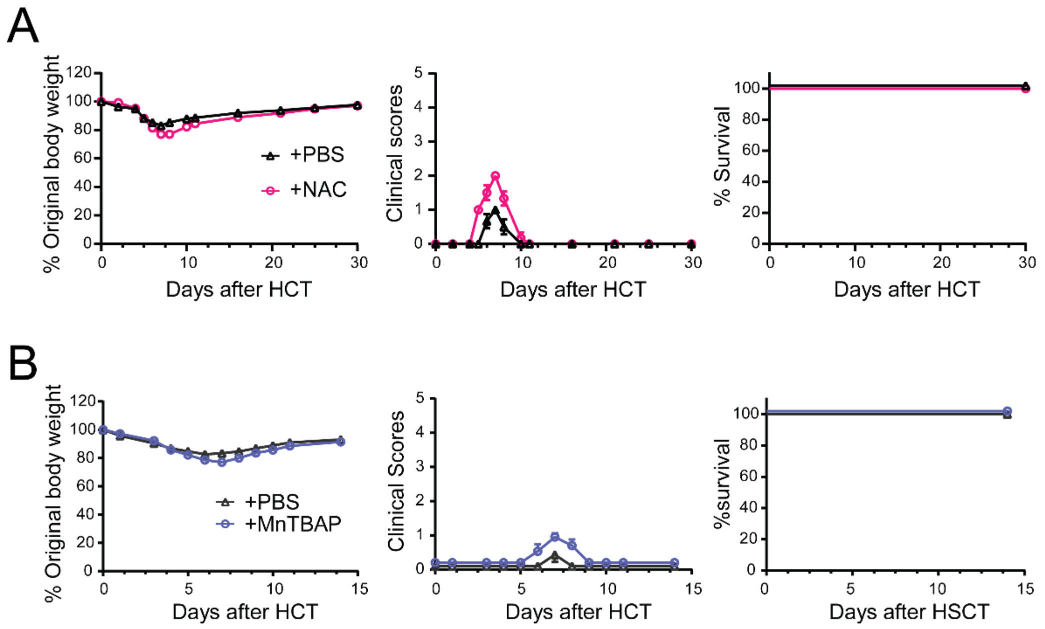

Figure S12. Antioxidant reagents did not reverse prevention of GVHD by Pan-Stat3 deficiency in donor T cells. Lethally irradiated BALB/c recipients were engrafted with TCD-BM cells (5 x 10^6) and CD90.2$^+$ T cells (2.5 x 10^6) from STAT3$^{-/-}$ C57BL/6 donors. **(A)** Recipients were given i.p. injection of NAC (2.5 mg/mouse), **(B)** MnTBAP (250 µg/mouse) on days 0, 2, 4, and 6. Plots of %original body weight, clinical GVHD score, and %survival curve are shown. *n* = 6 from 2 independent experiments.

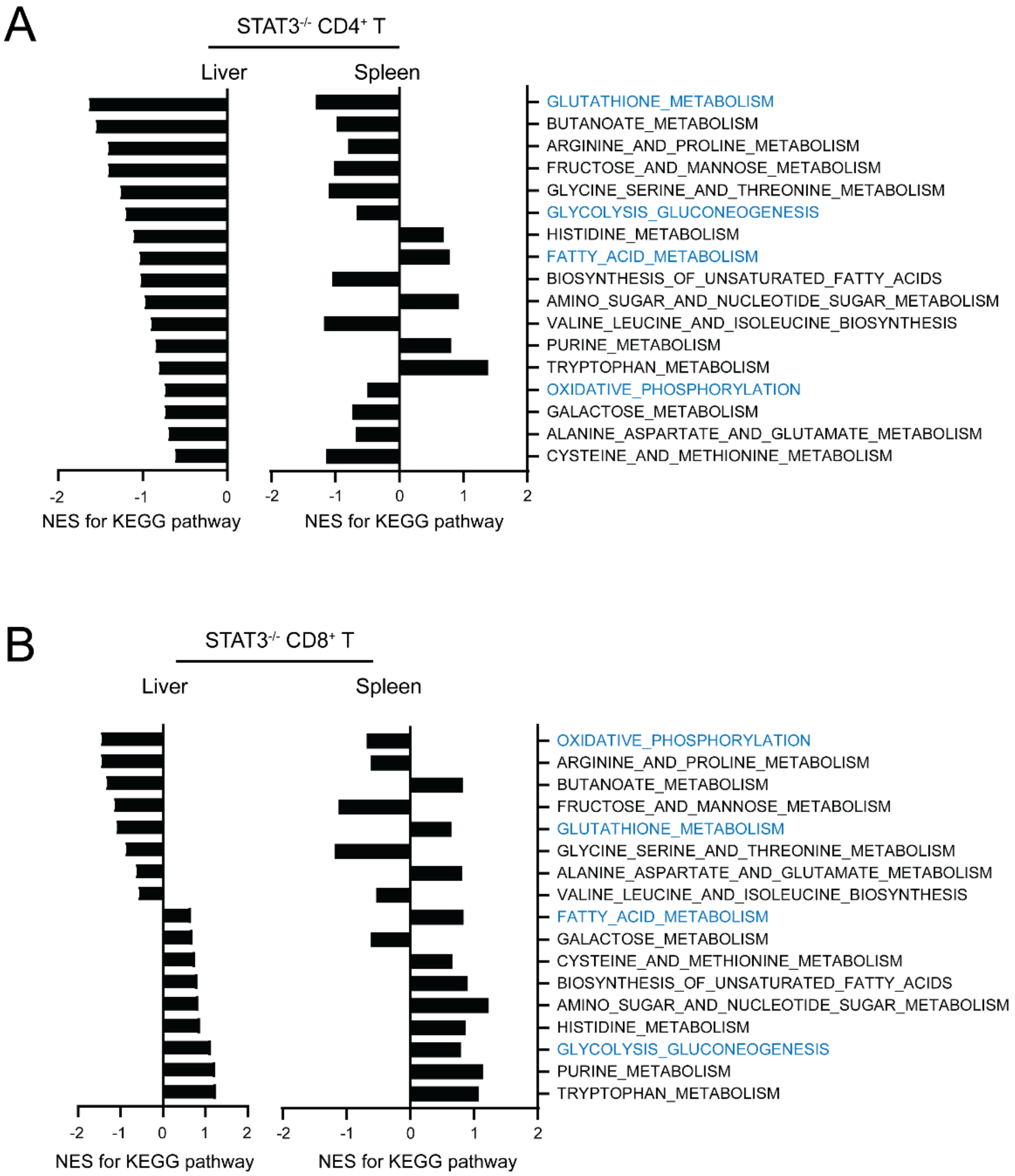

Figure S13. Comparison of NES of KEGG pathways of spleen and liver STAT3$^{-/-}$ T cells. These patterns are adopted from Figures 6A and 8B as well as Fig. S7A. **(A)** NES of KEGG Pathway activity of CD4^{+} T cells, setting WT CD4^{+} T cells as the reference. **(B)** NES of KEGG Pathway activity of CD8^{+} T cells, setting WT CD8^{+} T cells as the reference.

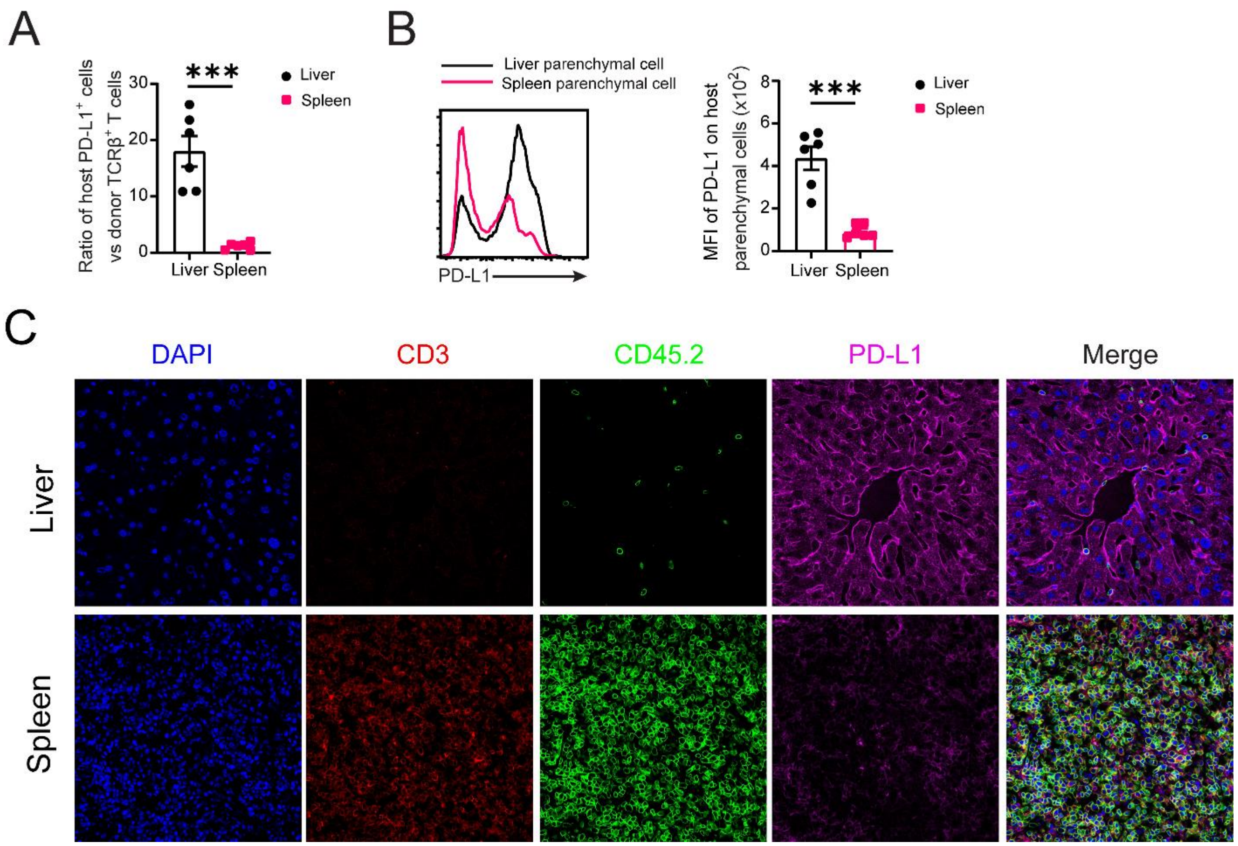

Figure S 14. Comparing ratios of CD45$^-$PD-L1$^+$ host-type parenchymal cells versus CD45$^+$TCRβ$^+$ or CD3$^+$ donor T cells in the liver and spleen of recipients. Irradiated BALB/c recipients were engrafted with TCD-BM (5 x 10^6) from WT C57BL/6 donors alone or with purified CD90.2$^+$ T cells (1 x 10^6) from spleens of STAT3$^{-/-}$ C57BL/6 donors. Day 6 after HCT, spleen and Liver were harvested for analysis. **[A-B]** Mononuclear cells from the liver and spleen of recipients were stained with anti-H2K^b, CD45, PD-L1, and TCRβ mAbs for flow cytometer analysis, with exclusion of dying and dead cells by Aqua staining. (A) Ratios of the percentage of H2Kb$^-$CD45$^-$PD-L1$^+$ host parenchymal cells versus H2Kb$^+$CD45$^+$ TCRβ$^+$ donor T cells in the liver and spleen are shown; (B) The MFI of PD-L1 on PD-L1$^+$H2Kb$^-$CD45$^-$ host-type parenchymal cells in the liver and spleen are shown, *n*=6 per group. Mean ± SEM combined from 3 replicate experiments. **(C)** immunofluorescent staining tissue slides of spleen and liver with DAPI, anti-CD3, CD45.2, and PD-L1. One representative is shown of 3 replicate experiments (Original magnification: 200x). *P* values were calculated by unpaired two-tailed Student t tests (***p <0.001).

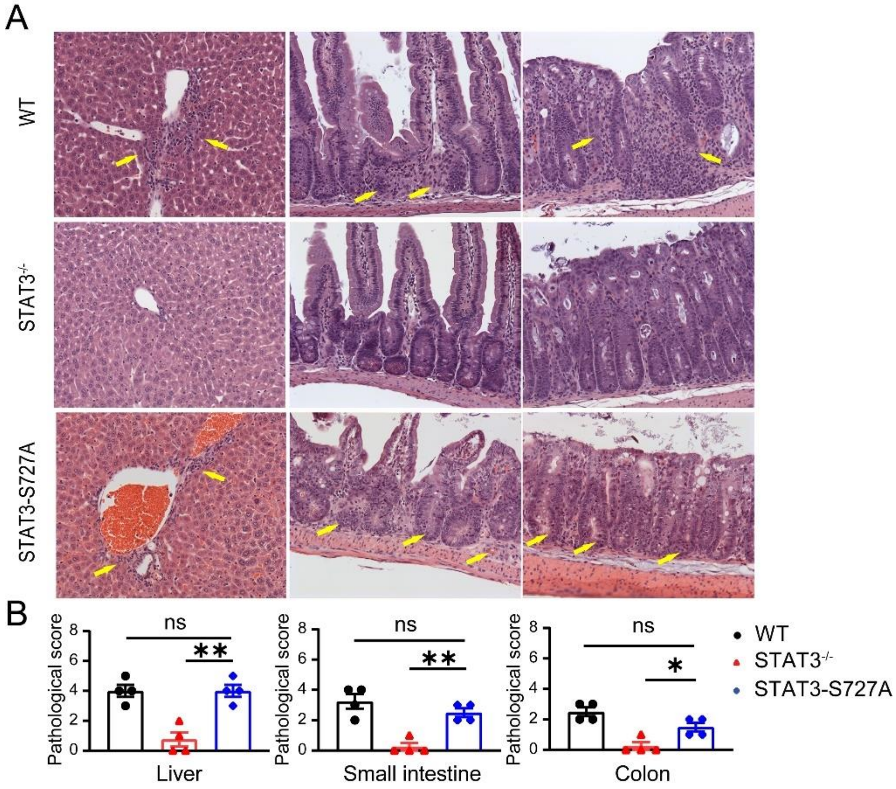

Figure S15. (Supplemental to Figure 9), Mito-STAT3 deficiency alone in donor T cells does not prevent GVHD. Lethally irradiated BALB/c recipients were engrafted with TCD-BM (5 x 10^6) from WT C57BL/6 donors and $CD90.2^+$ T cells (1 x 10^6) from WT or $STAT3^{-/-}$ or STAT3-S727A C57BL/6 donors. Histopathology of liver, small intestine, and colon was evaluated on day 6 after HCT. **(A)** Representative micrographic photos of liver, small intestine, and colon (arrows point to infiltrating T cells or tissue damage area, original magnification, ×200). **(B)** Means ± SEM of the GVHD scores of the liver, small intestine and colon are shown. *n* = 4 per group combined from 2 replicated experiments. *p* values were calculated by one-way analysis of variance. $^{ns}p \geq 0.05$, $^{*}p < 0.05$, $^{**}p < 0.01$.

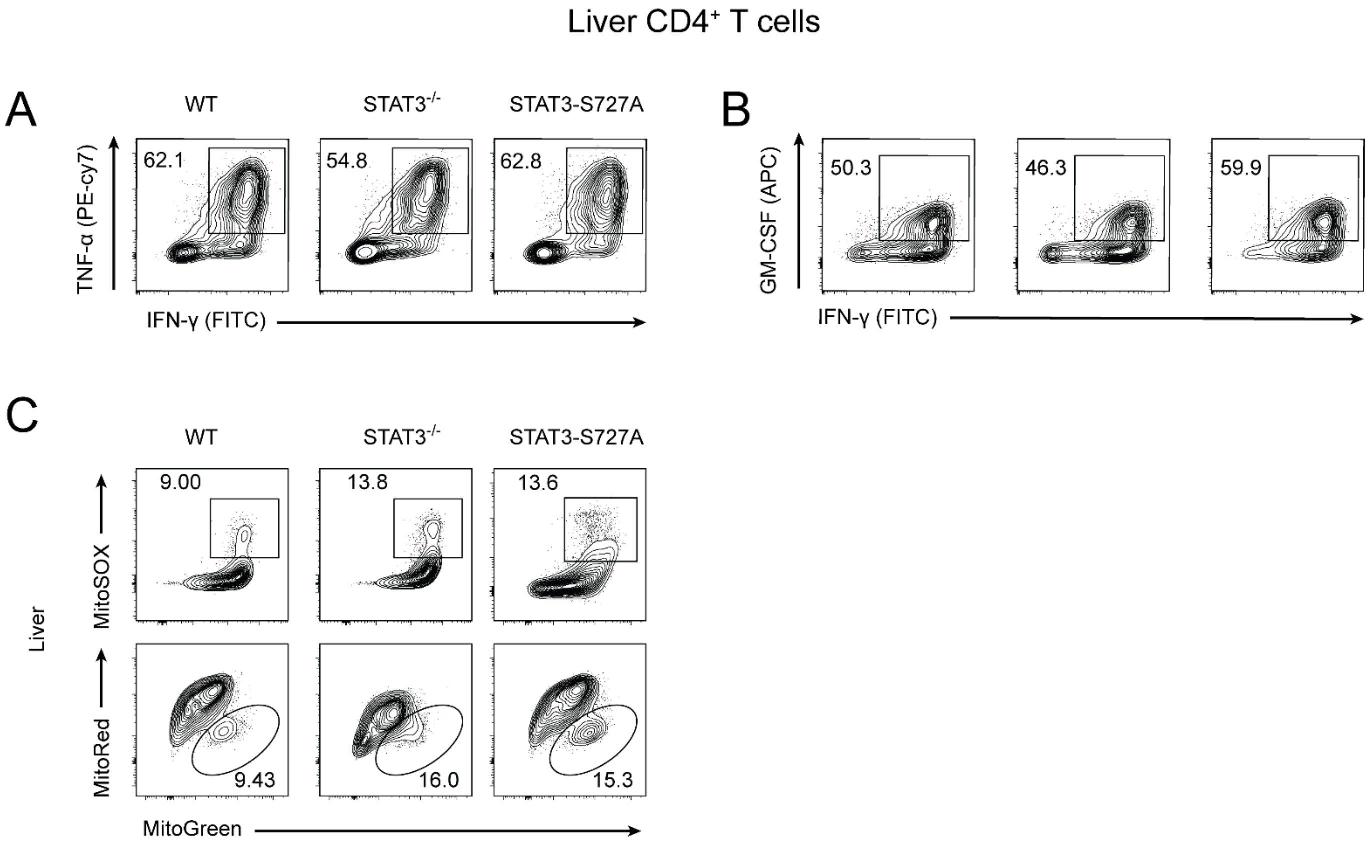

Figure S16 (Supplemental to Figure 9). Representative flow cytometry patterns of Fig. 9 (D & F).

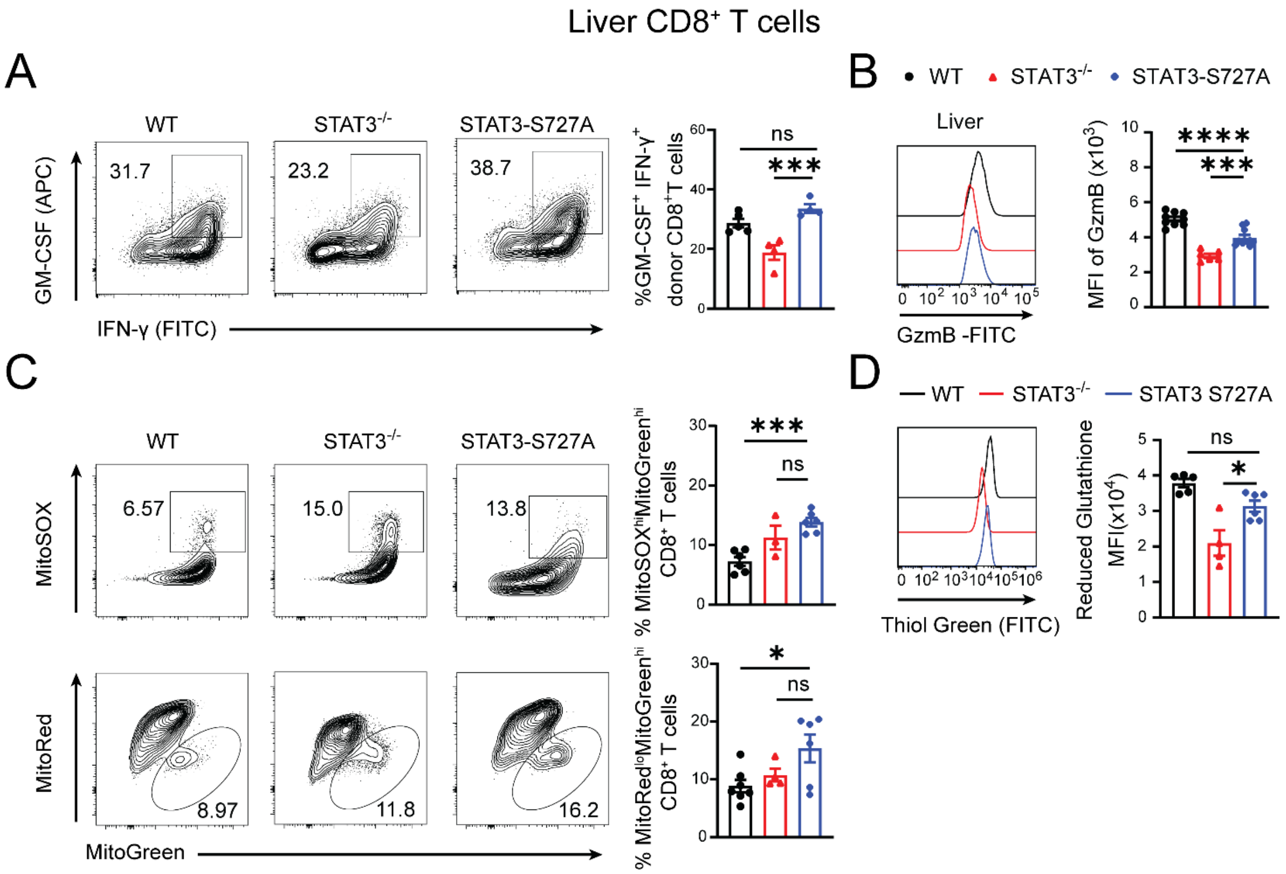

Figure S17. CD8⁺ T function in aGVHD target tissue is decreased by Pan-Stat3-deficiency but not by a loss of function Mito-Stat3 mutation. Lethally irradiated BALB/c recipients were engrafted with TCD-BM (5 x 10^6) from WT C57BL/6 donors and CD90.2⁺ T cells (1 x 10^6) from WT or STAT3$^{-/-}$ or STAT3$^{-/-}$PD-1$^{-/-}$ C57BL/6 donors. On day 6 after HCT, lymphocytes from liver were isolated for flow cytometry. **(A)** Representative flow cytometry patterns of GM-CSF/IFN-γ staining with %GM-CSF⁺/IFN-γ⁺ among CD8⁺ T cells are shown. **(B)** Means ± SEM of MFI of GzmB are shown. **(C)** Percent MitoSOX^{hi}MitoGreen^{hi} and MitoRed^{lo}MitoGreen^{hi} CD8⁺ T cells from different groups are compared. **(D)** Representative flow cytometry pattern and Means ± SEM of MFI of reduced Glutathione are shown. n = 4-9 per group combined from 2-3 replicated experiments. p values were calculated by one-way analysis of variance. $^{ns}p \geq 0.05$, $^{*}p < 0.05$, $^{**}p < 0.01$, $^{***}p < 0.001$, $^{****}p < 0.0001$.

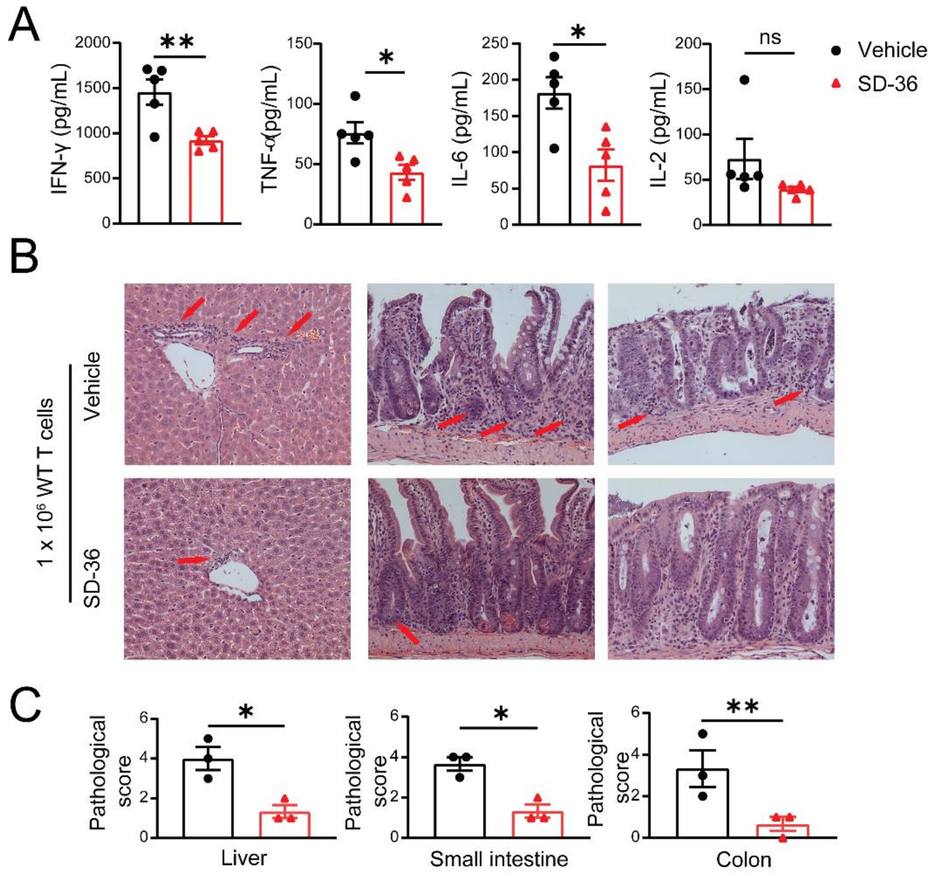

Figure S18 (Supplemental to Figure 10). Degradation of STAT3 in donor T cells prevents aGVHD. Irradiated BALB/c recipients were engrafted with TCD-BM (5 x 10^6) from WT C57BL/6 donors alone or with purified $CD90.2^+$ T cells (1 x 10^6) from spleens of WT C57BL/6 donors after culture in medium containing vehicle or 40μM SD-36 for 24 h in vitro. Recipients were treated with SD-36 (50 mg/kg, i.v.) or solvent on days 0 and 3 after HCT. **(A)** Serum concentration of IFN-γ, TNF-α, IL-6, and IL-2 on day 6 after HCT are shown. *n* = 4 per group combined from two replicated experiments. **[B-C]** Histopathology of liver (left), small intestine (middle) and colon (right) evaluated on day 6 after HCT. Representative micrographic photos are shown in (B) (arrows point at infiltrating T cells or tissue damage area, original magnification, 200x); the Means ± SEM of pathological scores are shown in (C). *n* = 3 per group combined from two replicate experiments. *p* values were calculated using unpaired two-tailed Student's t test for mean comparisons. $^{ns}p \geq 0.05$, $^*p < 0.05$, $^{**}p < 0.01$.

II. Supplemental Materials and Methods

Mice

BALB/c (H-2^d) and C57BL/6 (H-2^b) mice were purchased from the National Cancer Institute animal production program (Frederick, Maryland, USA). Stat3$^{fl/fl}$CD4-Cre C57BL/6 (H-2^b) breeder were kindly provided by Dr Hua Yu's lab at City of Hope National Medical Centre (Duarte, CA). PD-L1$^{-/-}$ BALB/c breeders were provided by L. Chen (Yale University, New Haven, Connecticut). PD-1$^{-/-}$ C57BL/6 (H-2^b) breeder mice obtained from Dr. Tasuku Honjo's laboratory (Kyoto University, Kyoto, Japan). PD-1$^{-/-}$ C57BL/6 (H-2^b) mice were crossed with Stat3$^{fl/fl}$C57BL/6 (H-2^b) mice to generate Stat3$^{fl/fl}$PD-1$^{-/-}$ CD4-Cre C57BL/6 (H-2^b) mice. Splenocytes from STAT3-S727A C57BL/6 (H-2b) and control mice were provided by Dr. M. Isbell (Department of Pathology, NYU Grossman School of Medicine, NY). Animal breeding and experiments were performed in separate specific pathogen-free rooms, and control and experimental mice were kept in separate cages in the same room at City of Hope Animal Research Center (ARC). In all, 12 light/12 dark cycle, temperatures of 68–75 °F with 30–70% humidity are used. All procedures were performed in the animal facility in compliance with a protocol approved by the City of Hope Institutional Animal Care and Use Committee (IACUC) under IACUC protocol 03008. All Mice were euthanized by CO_2 inhalation from compressed gas cylinders in compliance with all ethical regulations.

Induction and assessment of GVHD

WT BALB/c recipients were exposed to 850 cGy total body irradiation (TBI) with the use of a [$_{137}$Cs] source 8-10 hours before HCT, and then injected intravenously (i.v.) with splenocytes (5.0 x 10^6), Thy1.2$^+$ cells (1 x 10^6 or 2.5 x 10^6), and T cell-depleted BM (TCD-BM) cells (2.5 x 10^6) from C57BL/6 donors. Bone marrow T cell depletion was accomplished by using biotin-conjugated anti-CD4 and anti-CD8 mAbs, and anti-biotin Microbeads (Miltenyi Biotec, Germany), followed by passage through a MACS Column-based cell separation device (Miltenyi Biotec, Germany). Enrichment of Thy1.2$^+$ cells from spleen was accomplished by using mouse anti-CD90.2 microbeads (Miltenyi Biotec, Germany). The purity of enrichment was >98%, whereas the purity after depletion was >99%. The assessment and scoring of clinical signs of acute GVHD and clinical cutaneous GVHD has been described previously (1, 2)

Histopathology

Tissue specimens were fixed in formalin before embedding in paraffin blocks, sectioned, and stained with Hematoxylin and eosin (H&E). Slides were examined at 100x (liver) or 200x (small intestine and colon) magnification and visualized with Zeiss Observer II. Organ GVHD severity was blindly assessed according to a defined scoring system, as described previously (3). Liver GVHD was scored by the severity of lymphocytic infiltrate, proportion of involved tracts and severity of liver cell necrosis; the maximum score is 9. Gut GVHD was scored by mononuclear cell infiltration and morphological aberrations (e.g. hyperplasia and crypt loss), with a maximum score of 8.

Bioluminescent imaging

Mice were inoculated with luciferase-expressing BCL1 cells (BCL1/ Luc$^+$) by i.p. injection or luciferase-expressing BEL-1 ALL cells (ALL/Luc$^+$) (4) by i.v. injection on day 0 after HCT. For in vivo imaging of tumor growth, 200μl of 15mg/ml firefly luciferin was i.p. injected (Caliper Life Sciences, Hopkinton, MA), and mice were anesthetized for analysis of tumor cell burden by using an IVIS100 (Xenogen) and AmiX (Spectral) imaging system. Data were analyzed by using Amiview software purchased from Spectral Instruments Imaging (New York, NY).

Blockade of mouse PD-1, PD-L1 and CD25 in vivo

Anti-mouse PD-1 (BioXCell, Clone 29F.1A12, Rat IgG2a) and Anti-mouse PD-L1 (BioXCell, Clone 10F.9G2, Rat IgG2b) was administered via intraperitoneal (i.p.) injection at 200 ug/mouse every 2 days from day 4 to day 8 after HCT (Fig. 3). Anti-mouse CD25 (BioXCell, Clone PC-61.5.3, Rat IgG1) was administered via intraperitoneal (i.p.) injection at 200 ug/mouse every 2 days from day 0 to day 6 after HCT (Fig. S2). Control groups were given isotype-matched mAb (Rat IgG2a, Rat IgG2b or Rat IgG1) of irrelevant specificity.

Isolation of cells from spleen, liver, colon, and Small Intestine

Spleen and liver were mashed through a 70 μm cell strainer, and mononuclear cells (MNC) were isolated with 40% and 70% percoll. Colon and Small Intestine were cut first longitudinally and then laterally into pieces of approximately 0.5 cm length. Tissue pieces were incubated with 20 mL of predigestion solution (1× HBSS) without containing 5 mM EDTA, 5% fetal bovine serum (FBS), 1 mM DTT) for 20 minutes at 37°C under continuous shaking, then passed through 70 μm strainer, and MNC were isolated with 40% and 70% percoll.

Antibodies, FACS analysis, and FACS sorting

mAbs to H-2K^b (Clone: AF6-88.5), TCR β chain (Clone: H57-597), CD4(Clone: RM4-5), CD8 α (Clone: 53-6.7), TNF-α (Clone: MP6-XT22), GM-CSF (Clone: XMG1.2) were purchased from BD Pharmingen (San Diego, CA). H-2K^b (Clone: AF6-88.5), IFN- γ (Clone: XMG1.2). mAbs to CD4 (Clone: RM4-5), GM-CSF (Clone: MP1-22E9), Granzyme B (Clone: QA16A02) were purchased from Biolegend (San Diego, CA). mAbs to H2Kb (Clone: 25-D1.16), H-2K^b (Clone: AF6-88.5.5.3), CD4 (Clone: GK1.5), Fxop3 (Clone: FJK-16s), FR4 (Clone: eBio12A5), CD73(Clone: TY/11.8), CD98 (Clone: RL388), CD36 (Clone: HM36) were purchased from eBioscience (San Diego, CA). mAbs to Glut1 (Clone: EPR3915), CPT1A (Clone: 8F6AE9), and polyclonal anti-Rabbit IgG H&L (Catalog#: ab150077), Trx1 (Catalog#: ab150077) were purchased from Abcam (Cambridge, UK).

For *In vivo* BrdU labeling and Annexin V staining. On day 6 after HCT, T cell proliferation was measured with a single i.p. injection of BrdU (2.5 mg/mouse, 100 mg/g) 3h before tissue harvesting. Analysis of donor T cells for BrdU incorporation was performed according to the manufacturer's instructions (BD Pharmingen). For Annexin V staining, the percentage of Annexin V$^+$ cells was assessed by flow cytometry according to the manufacturer's instructions (ThermoFisher, catalog# 88-8007-72). For mitochondrial staining, cells were stained with MitoSOX, Mitotracker green, Mitotracker Red dye (ThermoFishe, catalog# M36008, M7514, M7512) according to manufacturer's protocol. Intracellular reduced glutathione was assessed by flow cytometry after staining with Thiol Green according to the manufacturer's instructions (Abcam, catalog# ab112132). Flow cytometry analyses were performed with BD LSRFortessa (Franklin Lakes, NJ), data were analyzed with FlowJo software (Tree Star, Ashland, OR). Cell sorting was performed with a BD FACS Aria SORP sorter (Franklin Lakes, NJ) at the City of Hope FACS facility. The sorted cells were used for RNA isolation.

CDR3 sequencing and analysis

Mouse TCR alpha and beta were amplified using Takara's SMARTer mouse TCR kit to prepare sequencing libraries, which were quantified and sequenced on an miSeq sequencer (Illumina) to generate PE300 reads. TCR sequencing data were aligned and processed using miXCR v3.0 with default settings to obtain TCR CDR3 clones. VDJTools v1.1.7 were used to generate a merged CDR3 clone abundance table for all samples include two batches and each batch combined from 3 mice and to calculate the diversity index.

RNA isolation and RNA sequencing

Each sample represents lymphocytes combined from 3 recipients. RNA from equal numbers of sorted H-2K^{b+}TCRβ^{+}CD4^{+} and H-2K^{b+}TCRβ^{+}CD8^{+} cells were extracted with the RNeasy Mini Kit (Qiagen, Hilden, Germany). Total RNA sequencing was performed and analyzed by the Integrative Genomics Core, City of Hope National Medical Center (Duarte, CA).

For RNA sequencing, RNA concentration was measured by NanoDrop 1000 (Thermo Fisher Scientific, Waltham, MA), and RNA integrity was determined with the use of a 2100 Bioanalyzer (Agilent Technologies, Santa Clara, CA). Libraries were constructed from 300 ng total RNA for each sample by using KAPA Stranded mRNA-Seq Kit (Kapa Biosystems, Wilmington, MA) with 10 cycles of PCR amplification. Libraries were purified using AxyPrep Mag PCR Clean-up kit (Thermo Fisher Scientific). Each library was quantified by using a Qubit fluorometer (Life Technologies, Carlsbad, CA), and the size distribution was assessed by using a 2100 Bioanalyzer (Agilent, Santa Clara, CA). Cluster generation was done according to the TruSeq SR Cluster Kit V4-cBot-HS (Illumina, San Diego, CA), and sequencing was performed on an Illumina® Hiseq 2500 (Illumina, San Diego, CA) instrument to generate 51 bp single-end reads. Quality control of RNA-Seq reads was performed using FastQC.

Gene Set Enrichment Analysis

Raw sequences were aligned to the mouse reference genome mm10 using STAR aligner v2.7, and the gene level expression of RefSeq annotation were summarized using Htseq-count v0.11. Raw counts were normalized by trimmed mean of M value (TMM) method and differentially expressed genes between groups were identified using the Bioconductor package “edgeR” v3.32.1. Pre-ranked gene set enrichment analysis (GSEA) was applied to examine the Hallmark and KEGG pathways, obtained from “msigbdr” package v7.4.1, that are significantly modulated using the “clusterProfiler” package v3.16.1. The gene list was ranked by -log10 (P value) with signs determined by logFC for each comparison for the GSEA analysis. Enrichment scores (ES) obtained by GSEA were used to compare different Hallmark and KEGG pathways at different time points after transplantation. P values were adjusted by the “BH” algorithm to generate false discovery rate (5).

Seahorse XF Real-Time ATP Rate Assay

Seahorse experiments were performed on sorted Thy1.2^{+} cells from GVHD recipients using Seahorse XF Real-Time ATP Rate Assay Kit (Seahorse Bioscience, catalog# 103592-100). Briefly, cells (0.1×10^{6} cells/well) were plated on poly-D-lysine-coated 96-well polystyrene Seahorse plates, equilibrated for 1h at 37 °C and assayed for Real-Time ATP accumulation after addition of oligomycin (1 μM), and antimycin A/rotenone (1 μM/0.1μM). The increased ATP availability of WT and STAT3$^{-/-}$PD-1$^{-/-}$ Thy1.2^{+} cells were normalized in STAT3$^{-/-}$ Thy1.2^{+} cells.

Mixed lymphocyte reaction (MLR)

CD90.2^{+} T cells were purified from WT or Stat3$^{fl/fl}$CD4-Cre C57BL/6 spleens by using CD90.2 Microbeads (Miltenyi Biotec, Germany). Dendritic cells (DCs) purified from BALB/c spleens by CD11c Microbeads (Miltenyi Biotec, Germany) were exposed to 3000-3500 cGy irradiation. A total of 1.5×10^{5} CD90.2^{+} WT or STAT3$^{-/-}$ T cell labeled with or without CFSE (Fisher Scientific, catalog# C34554) were cultured with 1.5×10^{5} irradiated DCs for 96 h, respectively. MLRs were performed in 96-well flat-bottom plates in RPMI 1640 medium supplemented with 10% FBS, L-glutamine and penicillin/streptomycin. Cells were collected and stained for flow cytometry analysis CD8^{+} T cell at days 3 and CD4^{+} T cell at 4 after coculture. Supernatants were collected for INF-γ and TNF-α measurement by Cytometric Bead Array assay (BD bioscience, catalog# 560485).

Mitochondria Isolation

Mitochondrial isolation were performed on sorted Thy1.2^{+} cells from GVHD recipients according to the procedure in reference (6). Briefly, cells were homogenized to release mitochondria and other fractions, and then mitochondrial fractions were collected using differential centrifugation.

Antibodies, and Immunoblotting

mAbs to Phospho-Stat3-ser727 (Clone: D8C2Z), Lamin A/C (Clone: 4C11), GAPDH (Clone: D4C6R) and polyclonal anti-αTubulin (Catalog#: 2144), COX iv (Catalog#: 4844) were purchased from Cell Signaling Technology (Danvers, MA). Anti-Pan-STAT3 mAb (Clone: NL557) was purchased from R&D Systems (Minneapolis, MN). Anti-Myc mAb (Clone: 9E10) was purchased from Santa Cruz Biotechnology (Dallas, TX).

Cells were lysed by RIPA Buffer (Fisher scientific, #PI89900), resolved by 4–15% precast protein gels gel (Bio-Rad, catalog# 4561086), and transferred to a PVDF membrane (Fisher Scientific, catalog# 09-720-3). Membranes were blocked for 1 hour using 10% Skim Milk (VWR, catalog# 90002) in Tris-buffered saline with Tween 20 (TBST). The PVDF membranes were incubated with SuperSignal West Pico Chemiluminescent Substrate (Thermo Fisher Scientific, catalog# 34577) for 5 min, and films were developed using a X-ray film developer. Films were scanned by GS-900 Calibrated Densitometer (Bio-Rad Laboratories, Hercules, CA). Grayscale values were obtained with ImageJ v2.9.0. To perform the normalization calculations, signals from the protein of interest (Myc, pan-STAT3, mito-STAT3) were normalized by its Grayscale values vs housekeeping (GAPDH, COX iv) protein in each lane to yield the self-normalized expression signal. The self-normalized expression signals of the control lane (WT, before HCT) were used as the reference lane for the rest of the blot. Hence, the relative expression levels were determined for each lane by comparing the self-normalized expression signal from each lane vs the reference lane.

Immunofluorescence Staining

For liver and spleen PD-L1 staining, liver and spleen cryosections were stained with rabbit monoclonal anti-mouse CD3 (Clone: MA5-14524, Fisher Scientific), rat anti-mouse CD45.2 (Clone: 104, Biolegend) and rat anti-mouse PD-L1 (Clone: MIH5, Fisher Scientific) according to previous publications of our group(7). Images were acquired on a Confocal Zeiss LSM700 microscope at 200X magnification.

Degradation of STAT3 protein in vitro and in vivo.

In vitro, CD90.2^{+} T Cells were purified from WT, PD-1$^{-/-}$ C57BL/6 spleens by using CD90.2 Microbeads (Miltenyi Biotec, Germany). Cells were seeded in 6-well white plates and treated with SD-36 (MedChemExpress, catalog# HY-129602) or DMSO for 24 hours. In vivo, SD-36 was administered via intravenous (i.v.) injection at 50 mg/kg at days 0 (after irradiation, and while injecting T cells) and 3 after HCT.

Statistical Methods

Data were shown as mean ± SEM. Comparison of %Survival in groups was analyzed by log-rank test. Two group means comparison was analyzed by using an unpaired two-tailed Student t-test. For evaluation of three means, we use one-way ANOVA multiple comparisons. For evaluation two independent variables on a dependent variable, we use two-way ANOVA multiple comparisons (Prism version 7). Adjusted p-values in GSEA plots of MYC target V2 pathway were calculated by using the clusterProfile v3.16.1 and msigdbr v7.4.1 packages in R. Two-sided p-values less than 0.05 and adjusted GSEA p-values less than 0.25 were considered statistically significant.

References:

1. Ni X, Song Q, Cassady K, Deng R, Jin H, Zhang M, et al. PD-L1 interacts with CD80 to regulate graft-versus-leukemia activity of donor CD8+ T cells. *J Clin Invest.* 2017;127(5):1960-77.
2. Song Q, Wang X, Wu X, Qin H, Li Y, Riggs AD, et al. Tolerogenic anti-IL-2 mAb prevents graft-versus-host disease while preserving strong graft-versus-leukemia activity. *Blood.* 2021;137(16):2243-55.
3. Chakraverty R, Cote D, Buchli J, Cotter P, Hsu R, Zhao G, et al. An inflammatory checkpoint regulates recruitment of graft-versus-host reactive T cells to peripheral tissues. *J Exp Med.* 2006;203(8):2021-31.
4. Chen Z, Shojaee S, Buchner M, Geng H, Lee JW, Klemm L, et al. Signalling thresholds and negative B-cell selection in acute lymphoblastic leukaemia. *Nature.* 2015;521(7552):357-61.
5. Benjamini Y, and Hochberg YJJotRsssB. Controlling the false discovery rate: a practical and powerful approach to multiple testing. 1995;57(1):289-300.
6. Wieckowski MR, Giorgi C, Lebiedzinska M, Duszynski J, and Pinton P. Isolation of mitochondria-associated membranes and mitochondria from animal tissues and cells. *Nat Protoc.* 2009;4(11):1582-90.
7. Deng R, Hurtz C, Song Q, Yue C, Xiao G, Yu H, et al. Extrafollicular CD4(+) T-B interactions are sufficient for inducing autoimmune-like chronic graft-versus-host disease. *Nat Commun.* 2017;8(1):978.
